# Identification of MAP kinase pathways as therapeutic targets in gallbladder carcinoma using targeted parallel sequencing

**DOI:** 10.18632/oncotarget.16751

**Published:** 2017-03-31

**Authors:** Mengdan Li, Lihong Chen, Yiping Qu, Fang Sui, Qi Yang, Meiju Ji, Bingyin Shi, Mingwei Chen, Peng Hou

**Affiliations:** ^1^ Department of Endocrinology, The First Affiliated Hospital of Xi'an Jiaotong University School of Medicine, Xi'an 710061, P.R. China; ^2^ Department of Critical Care Medicine, The First Affiliated Hospital of Xi'an Jiaotong University, Xi'an 710061, P.R. China; ^3^ Key Laboratory for Tumor Precision Medicine of Shaanxi Province, The First Affiliated Hospital of Xi'an Jiaotong University, Xi'an 710061, P.R. China; ^4^ Department of Respiratory and Critical Care Medicine, The First Affiliated Hospital of Xi'an Jiaotong University, Xi'an 710061, P.R. China

**Keywords:** gallbladder carcinoma (GBC), targeted massively parallel sequencing, MAP kinase pathways, Wnt/β-catenin pathway, NF-κB pathway

## Abstract

The aim of this study was to profile somatic mutation spectrum in gallbladder cancers (GBCs), and determine the role of MAP kinase pathway in GBC by a series of *in vitro* and *in vivo* studies. We performed targeted massively parallel sequencing of DNA isolated from GBCs and matched blood from 14 GBC patients to search for mutations in 504 genes commonly altered in human cancers. We identified recurrent mutations enriched in several major signaling pathways including MAP kinase, Wnt/β-catenin and NF-κB pathways. Immunohistochemistry analysis further validated overactivation of MAP kinase and Wnt pathways in a panel of GBC samples. By treating GBC cells with MEK inhibitor trametinib, we found that trametinib not only dramatically inhibited the activity of MAPK/ERK pathway, but also blocked the Wnt/β-catenin signaling through decreasing β-catenin expression or suppressing nucleus translocation of β-catenin. Moreover, trametinib inhibited the proliferation of GBC cell in a dose- and time-dependent manner, induced GBC cell apoptosis, and inhibited GBC cell migration and invasion. Growth of xenograft tumors derived from GBC cell line NOZ in nude mice was also significantly inhibited by trametinib. Our data highlight the critical role of MAP kinase pathways in GBC pathogenesis, and may represent therapeutic targets for this cancer.

## INTRODUCTION

Gallbladder carcinoma (GBC) is the most common malignant tumor of the biliary tract, and the sixth most common form of digestive tract malignancy [1]. Although GBC is relatively uncommon malignancy worldwide, its incidence varies widely among different regions. In South America and Asia, there is high incidence and mortality rate [2, 3]. About 10% of patients who found at early-stage are suitable for surgical resection, which is the only potentially curative approach. However, due to the anatomical position and early metastases, most patients with GBC are found at an advanced stage, leading to poor prognosis [4].

GBC cells are apt to metastasis, but relatively little is understood about GBC compared with tumors of other related organs. Thus, a significant influx of new findings is required for this to be remedied. For example, the identification of susceptibility genes or oncogenic “driver” mutations, elucidation of the role of inflammation and an increased understanding of molecular events during gallbladder tumorigenesis should improve early diagnosis and treatment efficacy for GBC. It is clear that gallstones and chronic inflammation are two major risk factors leading to GBC development [5, 6]. Until now, there is relatively limited information about molecular alterations involved in pathogenesis and progression of GBC. *TP53* inactivation has been demonstrated to play a key and early role in GBC associated with gallstones and chronic inflammation [7–10]. There is increasing evidence that *KRAS* mutations are rarely found in GBC associated with gallstones [11, 12], but they are highly frequent genetic events in GBC associated with congenital abnormality of the pancreatic bile-duct junction (APBDJ) [13–16]. Of interest, a very recent study performed exome sequence from 57 GBC tumor-normal pairs and identified the most extensively mutated pathway is the ErbB signaling [17], suggesting that this pathway may play a critical role in GBC development.

In this study, we performed targeted massively parallel sequencing to examine the mutation profile in GBCs, and demonstrate the importance of MAP kinase pathways in gallbladder tumorigenesis.

## RESULTS

### Targeted gene sequencing identifies recurrent mutations in GBCs

**Figure 1:**
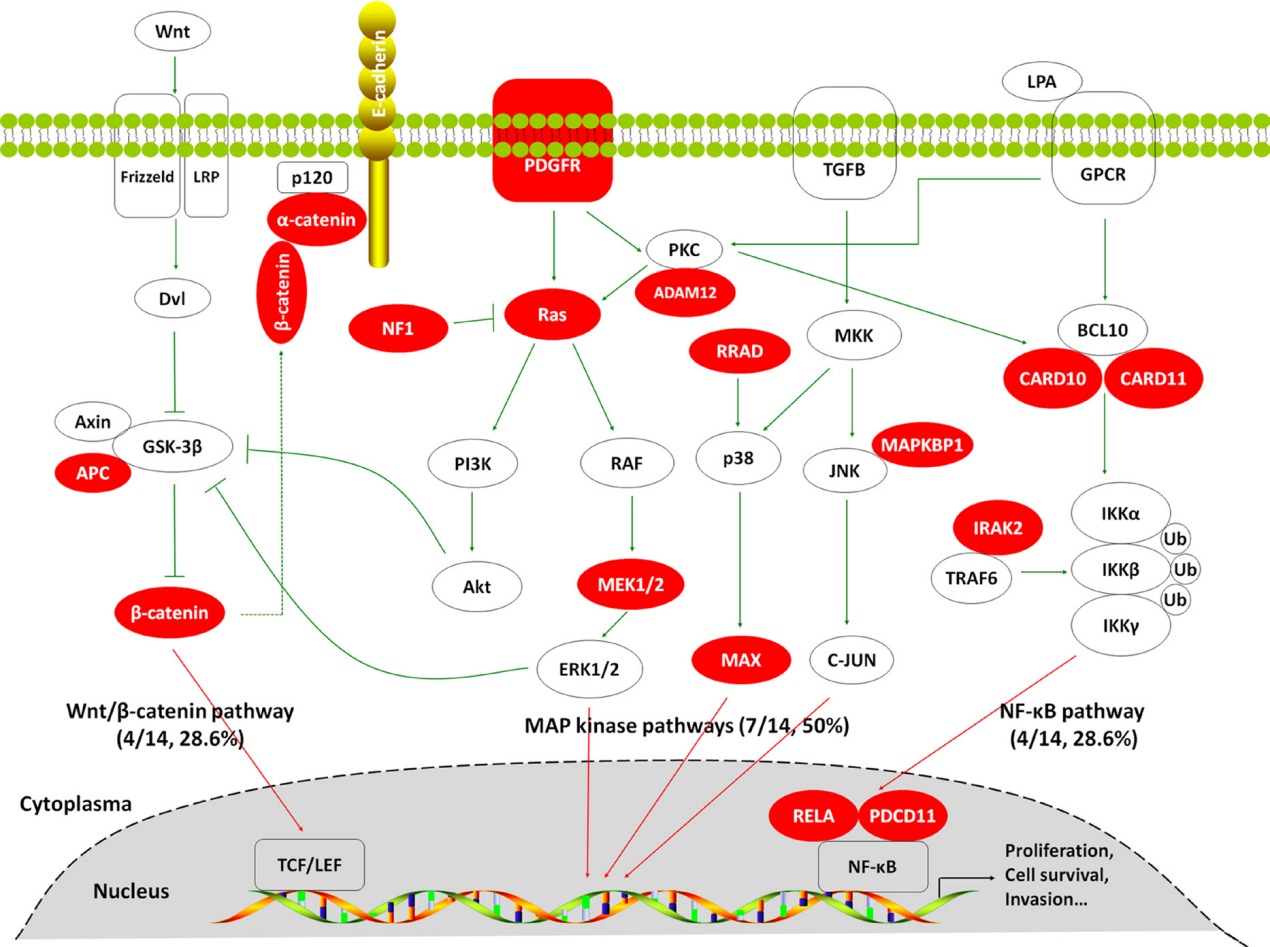
Somatic mutations identified by targeted massively parallel sequencing across 14 GBCs affecting the MAP kinase, Wnt/β-catenin and NF-κB signaling pathways Genes that are predicted to have gain or loss of function are depicted in red.

To investigate somatic mutation spectrum in GBCs, we performed targeted massively parallel sequencing of 504 genes, which are commonly mutated in human cancers in 14 pairs of GBC tissues and matched blood which proved by pathology and clinic. Through systematic analysis, we identified 63 somatic SNVs and 4 somatic insertions or deletions in 67 genes, which were predicted to probably alter protein-coding sequence or function (Supplementary Table 3). As expected, *TP53* gene was most frequently mutated in 7 of 14 (50%) GBCs. In addition, we surprisedly found that the MAP kinase signaling pathway-related genes exhibited high frequency of recurrent mutations (7/14, 50%) such as *PDGFR*, *ADAM12*, *KRAS*, *NF1*, *MAP2K1/MEK1* and *MAPKBP1* (Table 1 and Figure 1). Also shown in Table 1 and Figure 1, somatic mutations in several key genes on the Wnt/β-catenin pathway (including *APC*, *CCND1*, *CTNNA1* and *CTNNB1*) and NF-κB signaling pathway (including *CARD10*, *CARD11*, *IRAK1*, *PDCD11* and *RELA*) have also been identified, although none of these achieved statistical significance. Most of them are activating nonsynonymous mutations. These findings suggest the key role of these signaling pathways particularly MAP kinase pathways in GBC pathogenesis.

**Table 1:** Somatic mutations in the components of MAP kinase, Wnt/β-catenin and NF-κB Signaling pathways in GBCs

Genes	Mutation types	Allele change	Amino acid change	Case No.	Pathways
*ADAM12*	Nonsynonymous	Exon16: c.1619-7G > C	Splicing	T7	MAP kinase
*KRAS*	Nonsynonymous	Exon3: c.183A > T	p.Q61H	T16	MAP kinase
*MAPKBP1*	Nonsynonymous	Exon30: c.3722C > T	p.P1241L	T9	MAP kinase
	Nonsynonymous	Exon29: c.3704C > T	p.P1235L	T9	MAP kinase
*MAX*	Nonsynonymous	Exon3: c.202A > G	p.K68E	T36	MAP kinase
	Nonsynonymous	Exon4: c.229A > G	p.K77E	T36	MAP kinase
*NF1*	Nonsynonymous	Exon14: c.1641+1G >A	Splicing	T4,16,17	MAP kinase
	Nonsynonymous	Exon52: c.7676-2A>T	Splicing	T4,16,17	MAP kinase
	Nonsynonymous	Exon53: c.7739-2A>T	Splicing	T4,16,17	MAP kinase
	Nonsynonymous	Exon15: c.1645C > G	p.L549V	T4,16,17	MAP kinase
*PDGFRA*	Nonsynonymous	Exon17: c.2381A > G	p.D794G	T7	MAP kinase
*RRAD*	Nonsynonymous	Exon4: c.371-8C > T	Splicing	T30	MAP kinase
*APC*	Frameshift deletion	Exon16: c.4385_4388del	p.1462_1463del	T14	Wnt/β-catenin
	Frameshift deletion	Exon14: c.4331_4334del	p.1444_1445del	T14	Wnt/β-catenin
	Frameshift deletion	Exon17: c.4385_4388del	p.1462_1463del	T14	Wnt/β-catenin
*CCND1*	Nonsynonymous	Exon4: c.718G > A	p.D240N	T9	Wnt/β-catenin
*CTNNA1*	Stopgain SNV	Exon3: c.265G > T	p.E89X	T9	Wnt/β-catenin
*CTNNB1*	Nonsynonymous	Exon3: c.134C > T	p.S45F	T16,17	Wnt/β-catenin
*CARD10*	Nonsynonymous	Exon20: c.3077G > A	p.C1026Y	T7	NF-κB
*CARD11*	Nonsynonymous	Exon25: c.3260+3A>G	Splicing	T9	NF-κB
*IRAK2*	Nonsynonymous	Exon12: c.1511G > A	p.R504Q	T9	NF-κB
*PDCD11*	Nonsynonymous	Exon18: c.2524G > A	p.E842K	T16	NF-κB
*RELA*	Stopgain SNV	Exon3: c.115G > T	p.E39X	T14	NF-κB

### Activation of MAPK/ERK and Wnt/β-catenin signaling pathways in GBCs

**Figure 2:**
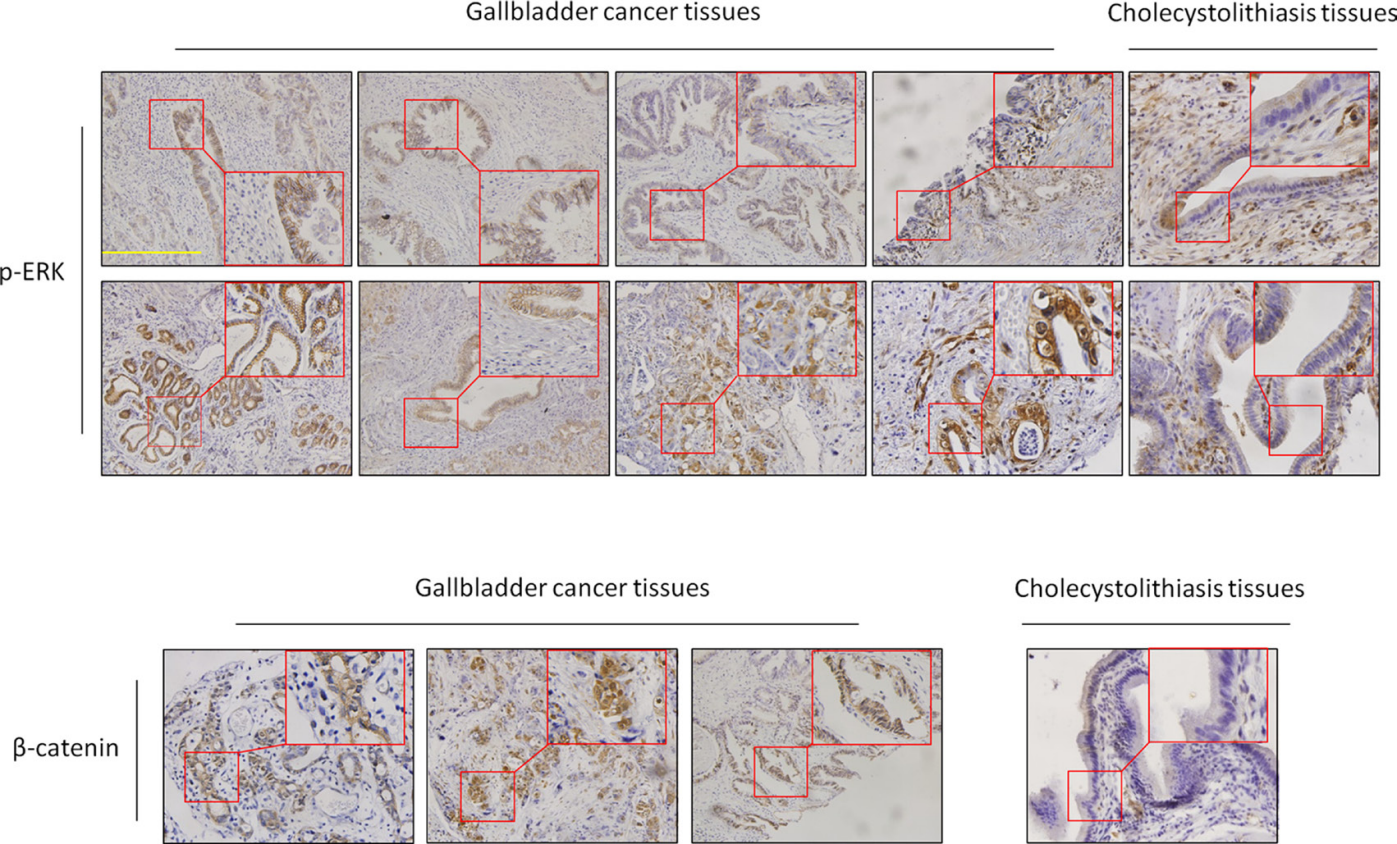
Activation of the MAPK/ERK and Wnt/β-catenin pathways in GBCs Immunohistochemistry analysis was performed to evaluate the levels of p-ERK1/2 and β-catenin in GBCs and normal gallbladder tissues. The positive staining was shown in a reddish-brown color. All sections were counterstained with Hematoxylin showing a blue color. The insert shows the magnified image of the area indicated by the red square. Scale bars, 200 μm.

To further verify the importance of MAP kinase and Wnt/β-catenin signaling pathways in GBCs, we performed immunohistochemistry assay to test the activities of these two pathways through detecting the expression levels of p-ERK1/2 (one of key markers for the activity of MAP kinase pathways) and β-catenin in 25 GBCs and 13 normal gallbladder samples (control subjects). As shown in Figure 2, expression levels of p-ERK1/2 and β-catenin in GBCs were significantly higher than these in control subjects. In particular, positive staining of p-ERK was found in 8 of 25 (32%) GBCs, but none in control subjects. These data suggest that the MAP kinase pathways such as the MAPK/ERK cascade plays a critical role in GBC development.

### MEK inhibitor trametinib inhibits the activities of the MAPK/ERK and Wnt/β-catenin signaling pathways in GBC cells

**Figure 3:**
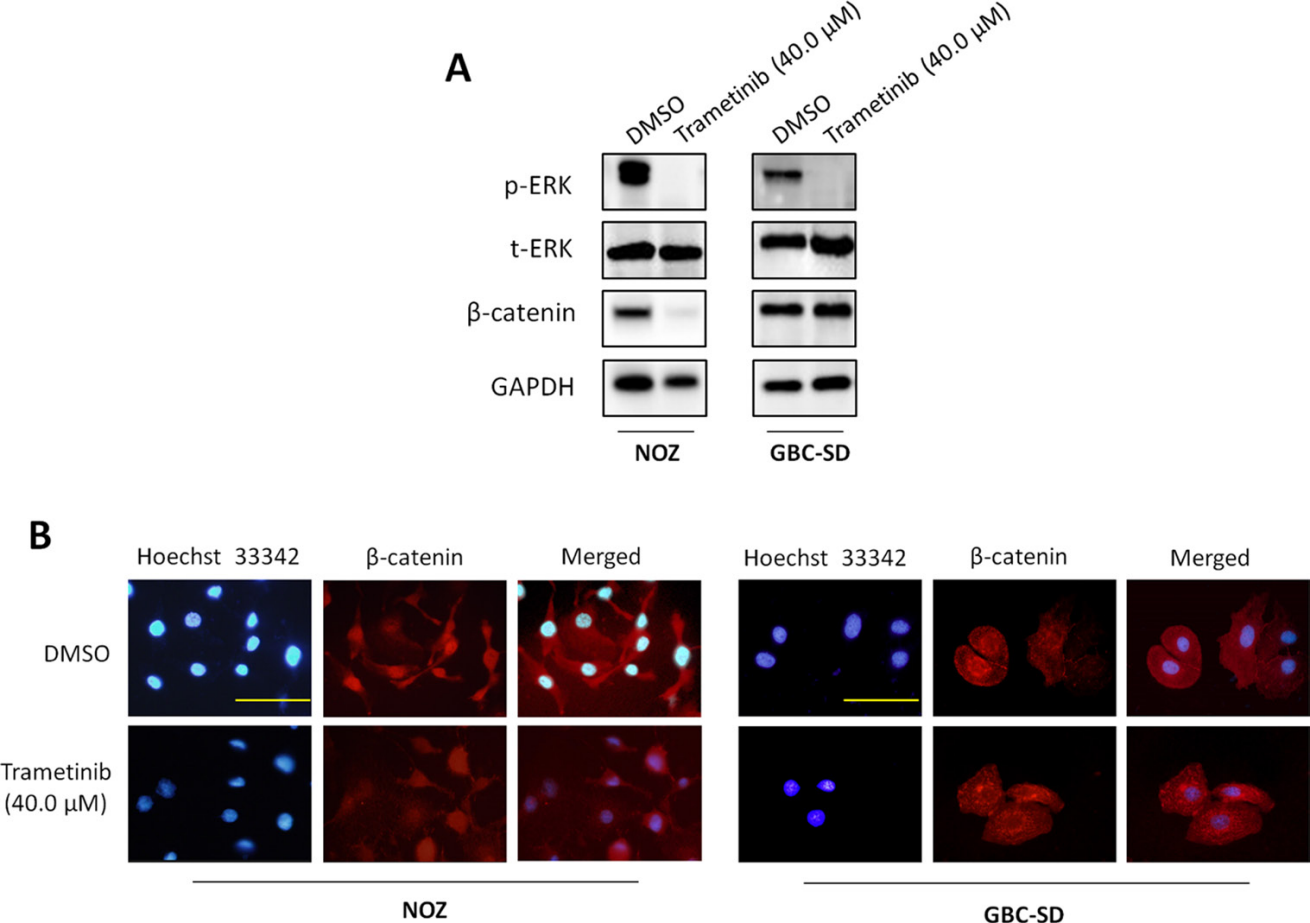
Inhibition of the activities of MAPK/ERK and Wnt/β-catenin pathways by trametinib NOZ and GBC-SD cells were treated with vehicle control (DMSO) or 40 μM trametinib for 48 h. Cell lysates were collected and subjected to Western blot and immunofluorescence assays. (**A**) The antibodies against phospho-ERK (p-ERK), total ERK (t-ERK) and β-catenin were used to determine the effect of trametinib on the levels of p-ERK and β-catenin in the indicated cells. GAPDH was used as a loading control. (**B**) Immunofluorescence assay was used to investigate the effect of trametinib on subcellular localization of β-catenin in NOZ and GBC-SD cells. Red color represents nuclear and cytoplasmic staining of β-catenin, and blue color represents Hoeschst 33342 staining for nuclei. Scale bars, 200 μm.

It is the fact that the MAPK/ERK pathway plays a fundamental role in tumorigenesis, and is activated by multiple cell surface receptors including receptor tyrosine kinases (RTKs), G protein-coupled receptors and cytokine receptors [18]. Thus, the kinases in this pathway have been heavily pursued as potential targets for cancer therapy [18–20]. MEK1/2 are key molecules in the MAPK/ERK signaling. Their inhibitors have recently been approved or are currently undergoing clinical trials, and exhibited potent antitumor activity [19, 20]. In this study, to determine the role of the MAPK/ERK pathway in GBC, we treated GBC cell lines NOZ and GBC-SD with MEK inhibitor trametinib (also known as GSK1120212), an orally available, potent, specific inhibitor of MEK1/2 activity with a long half-life period. As shown in Figure 3A, trametinib strongly decreased phosphorylation levels of ERK1/2 as compared to the control. Moreover, we found that trametinib also decreased the expression of β-catenin in NOZ cells as compared to the control. Although trametinib did not decrease expression of β-catenin in GBC-SD cells, we found that nuclear accumulation of β-catenin was dramatically inhibited when cells were treated with trametinib as compared to the control (Figure 3B). These findings are supported by a previous study that activated ERK serves as a scaffold to hold p90RSK and GSK-3β in a complex to facilitate GSK-3β phosphorylation by p90RSK, leading to inactivation of GSK-3β and upregulation of β-catenin [21]. These observations further support the importance of the MAPK/ERK signaling in gallbladder tumorigenesis.

### Trametinib inhibits GBC cell proliferation and colony formation

**Figure 4:**
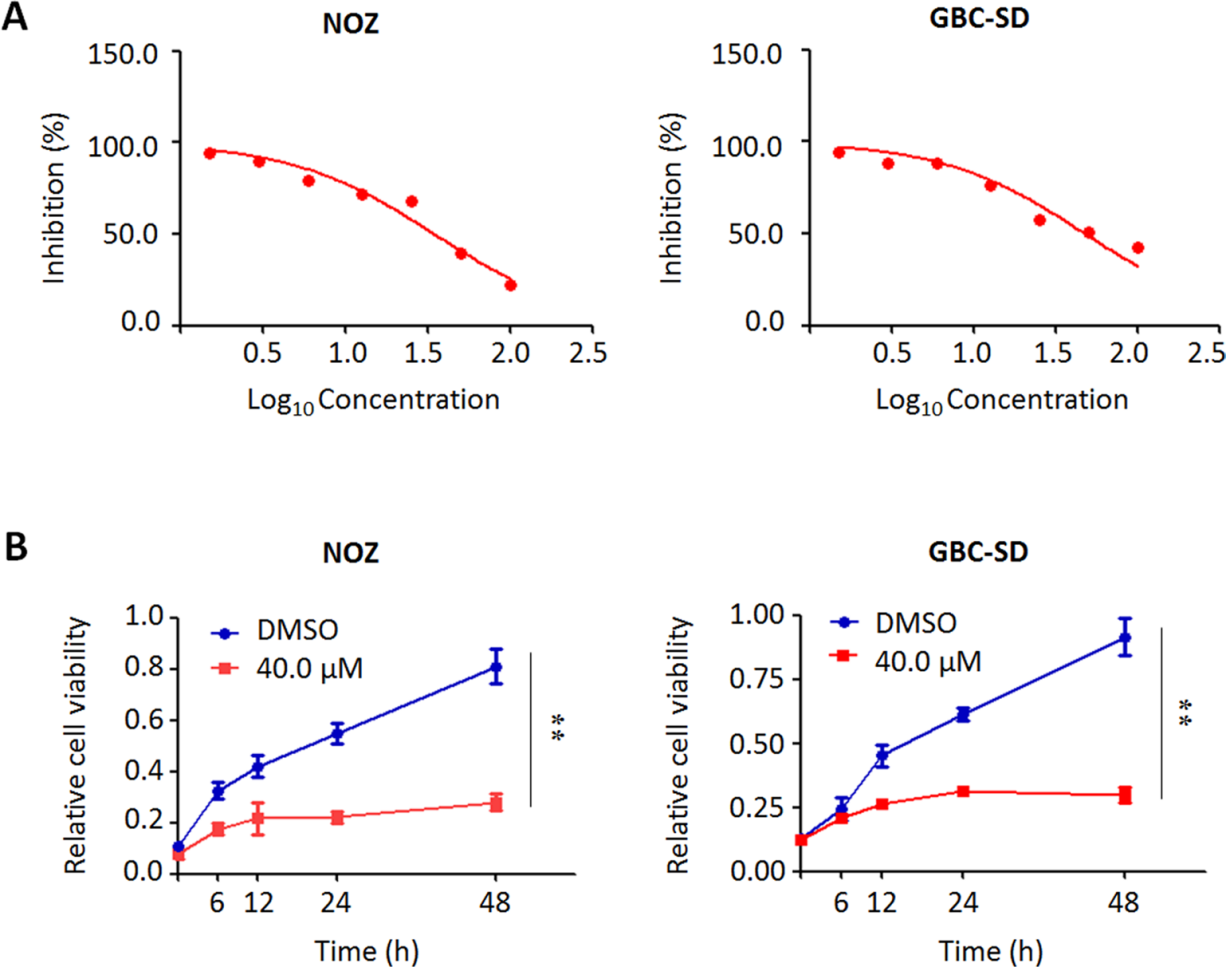
Inhibition of the proliferation of GBC cells by trametinib (**A**) NOZ and GBC-SD cells were treated with different doses of trametinib for 48 h. The MTT assay was performed to evaluate cell growth ability. (**B**) Time-course of cell proliferation was similarly measured by MTT assay in each cell line treated with vehicle control (DMSO) or 40 μM trametinib at the indicated time point. Statistically significant differences were indicated: **P < 0.01.

**Figure 5:**
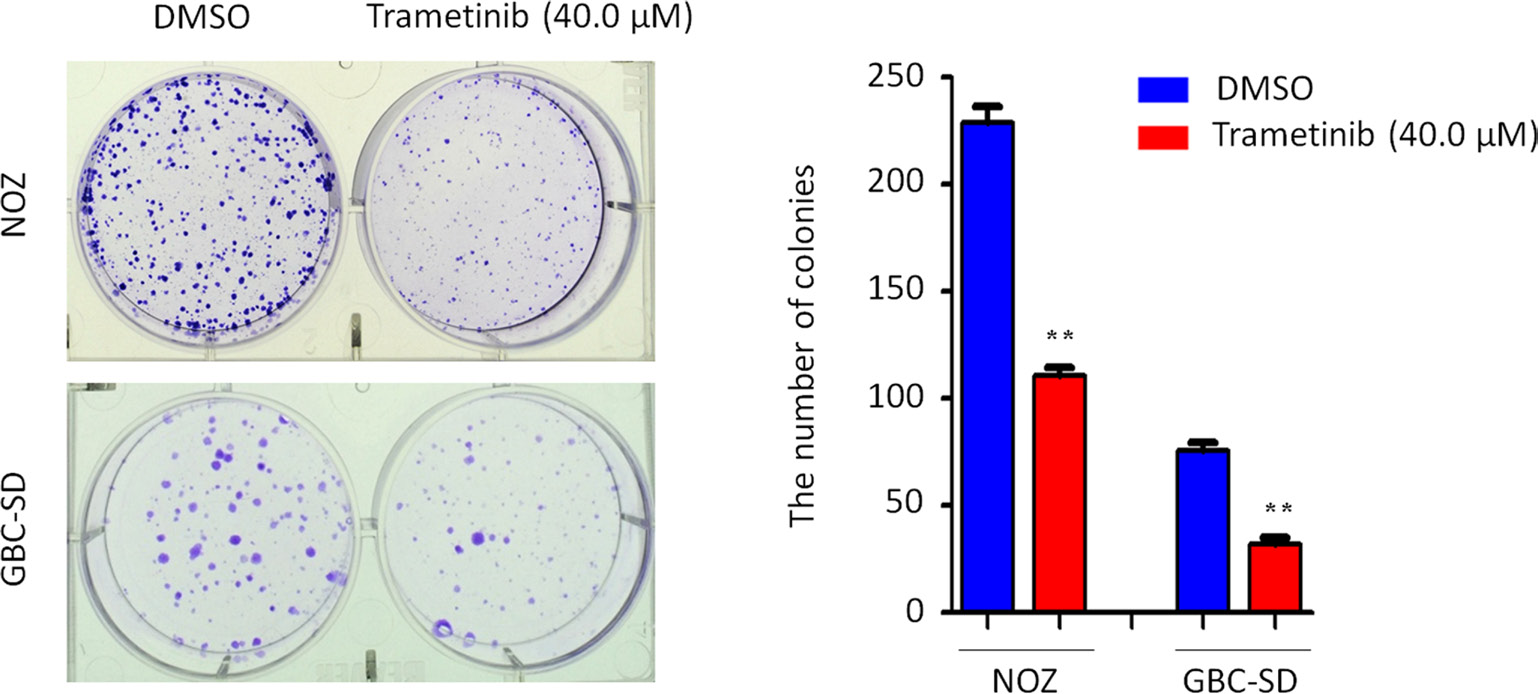
Inhibition of colony formation of GBC cells by trametinib Representative images of colony formation in NOZ and GBC-SD cells treated with vehicle control (DMSO) or 40 μM trametinib were shown in left panel. Quantitative analysis of colony numbers was shown in right panel. Data were presented as mean ± SD of values from three different assays. Statistically significant differences were indicated: **P < 0.01.

We performed the MTT assay to examine the dose and time course of the effect of trametinib on cell proliferation in GBC cells. As shown in Figure 4A, we found that trametinib significantly inhibited cell proliferation in a dose-dependent manner. In addition, we also test the effect of iCRT-14, a potent inhibitor of β-catenin-responsive transcription, on GBC cell proliferation. The results showed that iCRT-14 also dramatically inhibited cell proliferation in a dose-dependent manner (Supplementary Figure 1), further supporting that Wnt/β-catenin pathway may play a role in the pathogenesis of GBC. Next, we analyzed time-dependent response of GBC cells to trametinib. As shown in Figure 4B, trametinib significantly inhibited the proliferation of NOZ and GBC-SD cells at the indicated concentration and time points. Colony formation assay further confirmed the inhibitory effect of trametinib on cell growth. After treatment of trametinib, the ability of colony formation significantly decreased as compared to the control (Figure 5).

### Trametinib induces GBC cell apoptosis

**Figure 6:**
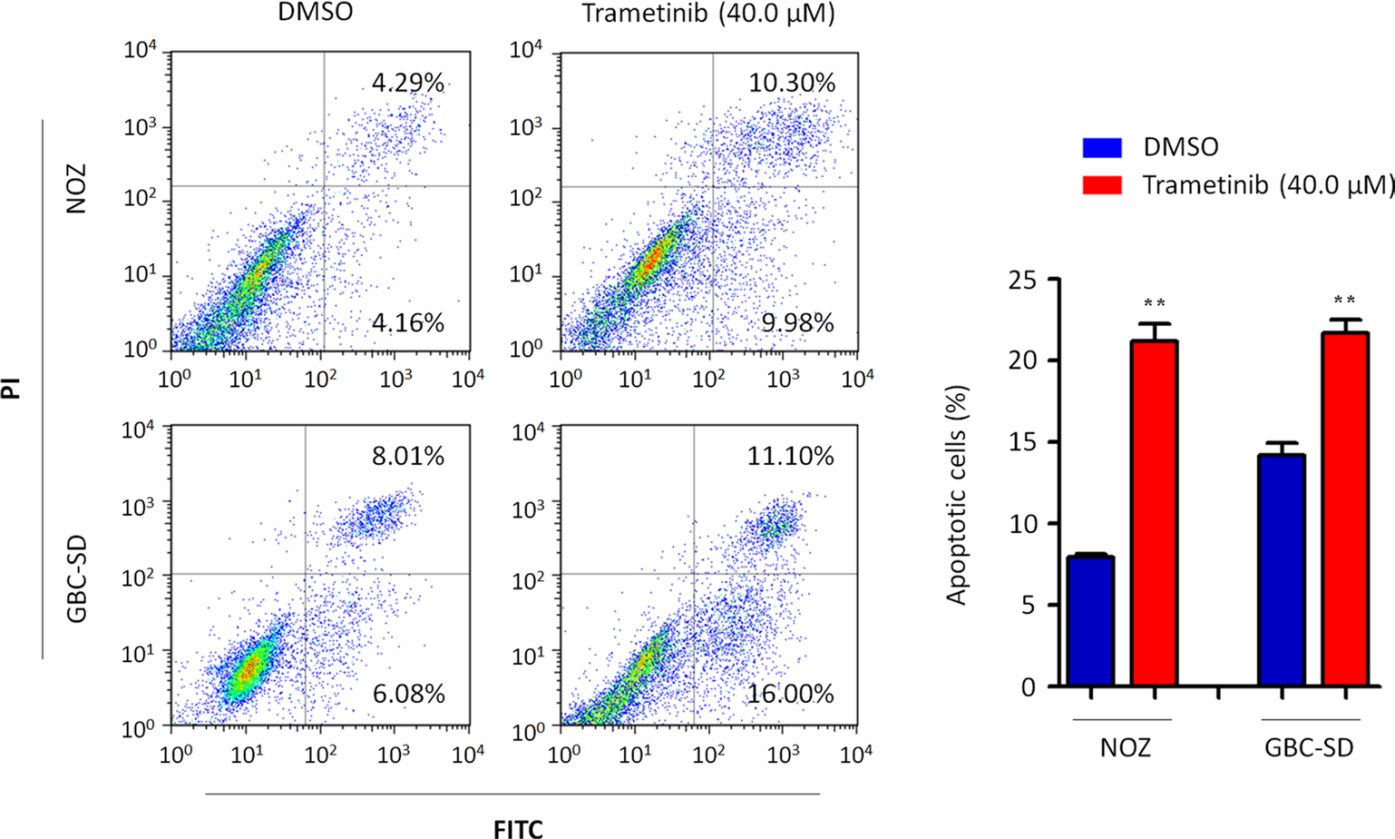
Induction of GBC cell apoptosis by trametinib Cell apoptosis was measured by flow cytometry analysis of Annexin V-FITC/PI double-labeled NOZ and GBC-SD cells treated with vehicle control (DMSO) or 40 μM trametinib for 48 h. The percentage of early apoptotic (bottom right quarter) and late apoptotic (top right) cells was presented in the figures. The data were presented as mean ± SD of values from three independent experiments. Statistically significant differences were indicated: **P < 0.01.

We also investigated the effect of trametinib on GBC cell apoptosis. As shown in Figure 6, both NOZ and GBC-SD cells treated with 40 μM trametinib for 48 h showed a dramatic increase in both early and late apoptosis as compared to the controls. The percentage of apoptotic cells was increased from 8.2 ± 0.2% to 21.0 ± 1.0% in NOZ cells (*P* = 0.005) and from 13.9 ± 0.4% to 23.7 ± 2.5% in GBC-SD cells (*P* = 0.024), respectively.

### Trametinib inhibits GBC cell migration and invasion

Given that tumor metastasis is a main cause of cancer-related death including GBC [1–3], we thus attempted to investigate the effect of trametinib on the the migrated ability and invasive ability of GBC cell in the present study. As shown in Supplementary Figure 2, there were significantly less migrated cells in GBC-SD cells treated with 40 μM trametinib than in the controls. In addition, the invasion assay showed that trametinib significantly inhibited the ability of GBC-SD cells to pass through the Matrigel-coated membrane. Taken together, our data suggest that trametinib inhibits the metastatic potential of GBC cells.

### Trametinib inhibits xenograft tumor growth

**Figure 7:**
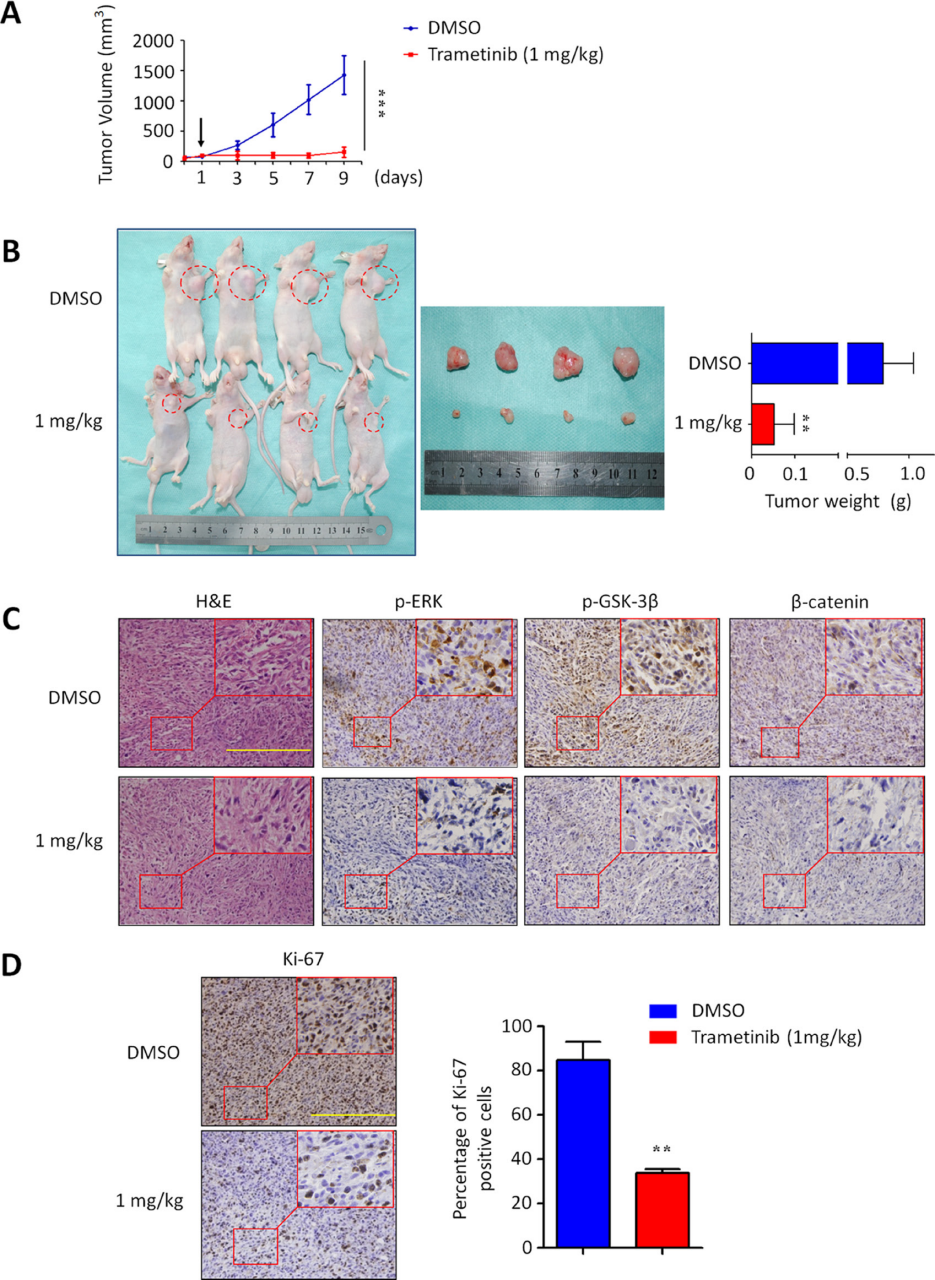
Inhibition of xenograft tumor growth by trametinib (**A**) Time course of NOZ cell-derived xenograft tumor growth, measured as tumor volume in mice treated with vehicle control or trametinib, 1 mg/kg orally, at the indicated time point. Data were presented as mean ± SD (*n* = 4/group). Arrow represents time point of tumor cell injection. (**B**) Shown is representative picture of tumor growth in mice treated with vehicle control (DMSO) and the indicated dose of trametinib (left and middle panels). Bar graphs represent mean of the tumor weight from control and trametinib-treated mice (right panel). Data were presented as mean ± SD (*n* = 4/group). (**C**) Tumor sections were subjected to immunohistochemistry using specific primary antibodies, including p-ERK, p-GSK-3β and β-catenin. (**D**) Shown is representative Ki-67 staining of xenograft tumors from control and trametinib-treated mice. Scale bars, 200 μm. Bar graphs represent mean ± SD of the numbers of Ki-67-positive cells from 5 microscopic fields in each group. Data were presented as mean ± SD. Statistically significant differences were indicated: **P < 0.01; ***P < 0.001.

Given *in vitro* inhibitory effect of trametinib on GBC cell growth, we supposed that trametinib has the potential to effectively treat GBC *in vivo*. Thus, we next tested the effect of trametinib on the growth of xenograft gallbladder tumors derived from NOZ cells in nude mice. As shown in Figure 7A, NOZ-derived xenograft tumors progressively grew in the control mice, whereas the tumors grew more slowly in the mice treated with trametinib at the indicated dose. By the end of the experiments, the tumors were isolated and weighted. As shown in Figure 7B, the mean tumor weight was significantly lower in trametinib-treated mice as compared to control mice (*P* = 0.029). To further validate the inhibitory effect of trametinib on the activities of the MAPK/ERK and Wnt/β-catenin signaling pathways, tumor sections were subjected to immunohistochemistry analysis using specific primary antibodies, including p-ERK1/2, p-GSK-3β and β-catenin. As shown in Figure 7C, the levels of p-ERK, p-GSK-3β and β-catenin were dramatically decreased in the tumors of trametinib-treated mice as compared to control tumors, suggesting that the activities of the MAPK/ERK and Wnt/β-catenin pathways were strongly inhibited by trametinib *in vivo*. To quantitatively evaluate the proliferation index of trametinib-treated tumors, the sections were stained by the Ki-67 antibody. As shown in Figure 7D, the number of Ki-67-positive cells was significantly decreased in the tumors of trametinib-treated mice as compared to control tumors (*P* = 0.005). Altogether, our data provide strong evidence that trametinib may be a potentially effective antitumor agent for GBC.

## DISCUSSION

GBC is one of the most deadly forms of human cancer, and is highly resistant to standard chemotherapy or radiotherapy [1]. However, knowledge of molecular and genetic mechanisms involved in GBC remains incomplete because of its relative rarity. In this study, our findings offer insights into the somatic mutational profile in GBCs and emphasize the key role of the MAP kinase signaling pathways in gallbladder tumorigenesis.

In fact, changes in MAP kinase pathways have been found in GBCs [3]. For example, the activation of *RAS* genes (including *KRAS*, *HRAS* and *NRAS*) caused by somatic mutations has been reported in GBCs and is well-documented to involved in multiple downstream signaling patways including the MAP kinase pathways [11–17]. Moreover, oncogenic *BRAF* mutations are thought to be even more important than mutations in *RAS* genes for activating the MAP kinase pathways in certain cancers particularly melanoma and thyroid cancer [22, 23]. A previous study examined the mutational status of *BRAF* gene in 21 GBCs, and identified *BRAF* mutations in 7 of 21 (33%) GBCs [24]. In addition, there was a mutual exclusivity between *BRAF* mutations and *RAS* mutations in these cases, implying that *BRAF* may play an important role in the pathogenesis of GBC. However, we did not find *BRAF* mutations in GBCs in this study. One possibility is that there are limited number of samples in this study.

Acting as receptors for growth factors, cytokines, hormones and other signaling molecules, receptor tyrosine kinases (RTKs) are a large and diverse group of transmembrane proteins and they can activate their downstream signaling pathways including MAP kinase pathways through ligand-induced dimerization [25, 26]. Aberrant constitutive activations of components of these pathways have been found in multiple human cancers including GBC, leading to increased proliferation, survival and metastasis of cancer cells including GBC [3, 17]. For example, the *ERBB2*/*HER2*, a key member of RTK family, plays an important role in tumorigenesis [27]. Its overexpression has been observed in 33–64% of GBCs [28–30], and genomic amplification has also been found in 70% of GBCs [28]. To be consistent with these findings, overexpression of ERBB2 in gallbladder epithelium leads to the development of adenocarcinoma in 100% of transgenic mice by 3 months of age through activating MAP kinase pathways [29]. In addition, a recent study reported frequent somatic mutations of *ERBBs* (including *EGFR*, *ERBB2*, *ERBB3* and *ERBB4*) and their downstream genes [17]. These observations suggest the key role of ErbB signaling pathway in GBC pathogenesis.

The MAP kinase pathways are thought to be the most important downstream signaling cascades of ErbB receptors, which regulate fundamental cellular processes such as cell proliferation, differentiation, migration and cell death [32]. The MAP kinases are divided into three main families, including ERKs (extracellular signal-regulated kinases), JNKs (Jun amino-terminal kinases) and p38/SAPKs (stress-activated protein kinases) [32]. Of them, the MAPK/ERK pathway is the best investigated of the mammalian MAP kinase pathways, and is aberrantly activated in about one-third of human cancers [33]. Many genetic alterations such as overexpression or activating mutations of RTKs, persistent paracrine or autocrine production of activating ligands, *RAS* and *BRAF* mutations lead to persistent activation of this pathway, contributing to tumorigenesis [33].

Given that MEK1/2 are exquisitely specific activators of ERK1/2 and their strategic position in the MAPK/ERK (RAS-RAF-MEK-ERK) pathway, they are thus considered as potentially drug targets in cancer. Until now, a number of MEK1/2 inhibitors have been developed and some of them have been tested clinically or currently undergoing clinical trials including biliary cancers (BCs) [20, 34]. There is promising evidence that selumetinib (AZD6244) may have potent activity in treating BCs, since 56% of patients obtained prolonged stable disease with no apparent side effects [34]. To date, trametinib is the first MEK1/2 inhibitor approved by the US Food and Drug Administration (FDA) [35]. In the present study, we evaluated *in vitro* and *in vivo* anti-tumor effect of trametinib in GBC. The results showed that trametinib dramatically inhibited GBC cell proliferation, colony formation, migration, invasion and tumorigenic potential in nude mice, and induced GBC cell apoptosis, suggesting that trametinib is an effective anti-tumor agent for GBC.

## MATERIALS AND METHODS

### Study subjects and DNA extraction

GBC samples and matched peripheral blood from 14 patients (Supplementary Table 1) for targeted sequencing, and paraffin-embedded gallbladder tissues from 25 patients with GBC and 13 patients with cholecystolithiasis for immunohistochemistry (IHC) were collected from the First Affiliated Hospital of Xi'an Jiaotong University between 2011 and 2013 under institutional review board approval and with documented informed consent. All patients did not receive chemotherapy and radiotherapy before the surgery, and all sections were histologically examined by a board-certified pathologist to ensure the cases with estimated carcinoma content of > 70%. Genomic DNA was extracted using standard phenol/chloroform protocol.

### Targeted gene sequencing

A total of 504 cancer-related genes were presented in Supplementary Table 2, and targeted for capture and deep sequencing. Using the eArray system (Agilent Technologies, Santa Clara, CA), the capture was designed to include all of protein coding sequences (CDSs) and most of the untranslated regions of these genes. In accordance with the manufacturer's protocol, genomic DNA was fragmented by the NEBNext dsDNA Fragmentase (New England Biolabs, Ipswich, MA), and daptor-ligated library was constructed using a Agilent SureSelect library kit (Agilent Technologies, Santa Clara, CA). Targeted sequence enrichment was performed using the Agilent SureSelect Target Enrichment Kit (Agilent Technologies, Santa Clara, CA) according to the manufacturer's instructions. The enriched samples were sequenced via 2 × 100 paried-end sequencing using a Hiseq 2000 Sequencing system (Illumina, San Diego, CA). Illumina Sequencing Control v2.8, Illumina Off-Line Basecaller v1.8 and Illumina Consensus Assessment of Sequence and Variation v1.8 software (Illumina, San Diego, CA) were used to produce 100 bp sequence reads.

### Sequencing data processing and mutation calling

SAMtools was used to generate a binary sequence alignment map (BAM) file for paired GBC and matched blood samples for each case. Next, somatic single-nucleotide variations (SNVs), insertions or deletions were detected by comparing the BAM files of GBC and matched blood. The resulting reads were aligned to the human reference genome (hg19) using the Burrows-Wheeler Aligner with default parameters [36]. Variants were identified using the Genome Analysis Toolkit [37] and VarScan software [38]. Coverage analysis was determined using the CalculateHsMetrics tool (Picard). Reads that matched exonic regions, including exon-intron boundaries, were also analysed. Relevant mutations in all of the genes were subsequently prioritized manually.

### Immunohistochemistry (IHC)

Paraffin-embedded sections (5 μm) were deparaffinized using xylene and graded ethyl alcohol and then rinsed in water. Antigen retrieval was performed by boiling the slides in Tris-EDTA buffer in a microwave oven for 10 min and cooling at room temperature. The slides were then incubated with 3% H_2_O_2_ to quench endogenous peroxides in a humidified chamber, blocking with goat serum for 10 min, primary antibody overnight at 4°C, secondary antibody for 15 min, and streptavidin-HRP conjugate for 15 min at room temperature. The slides were finally incubated with DAB for indicated times at room temperature. “Positive” was defined as increased staining intensity relative to normal tissues, and “negative” was defined by no significant increase in staining over normal tissues. The following antibodies were utilized: anti-phospho-ERK1/2 (p-ERK1/2) (Bioworld Technology), anti-β-catenin (Abcam), anti-phospho-GSK-3β (p-GSK-3β) (Cell Signaling Technology) and anti-Ki67 (BD Pharmingen).

### Cell culture and reagents

Human GBC cell lines NOZ (K-ras mutant) and GBC-SD were provided by Dr. Yingbin Liu (Department of General Surgery, Xinhua Hospital affiliated to Shanghai Jiaotong University School of Medicine, Shanghai, P.R. China) and Kunming Cell Bank of The Chinese Academy of Science (Kunming, P.R. China), respectively. These cells lines were not passaged for more than 6 months after authentication by the cell bank. NOZ and GBC-SD cells were cultured at 37°C in William's E medium or DMEM with 10% fetal bovine serum (FBS) (Invitrogen Technologies, Inc, CA), respectively.

In some experiments, cells were treated with MEK inhibitor trametinib (GSK1120212) and Wnt pathway inhibitor iCRT-14 as the indicated concentrations and times, and the medium and agent were replenished every day. GSK1120212 and iCRT-14 were purchased from Selleck Chemicals and Santa Cruz Biotechnology, respectively. Both of them were dissolved in dimethylsulfoxide (DMSO), aliquoted and stored at −80°C until use. The same volume of vehicle was used as control.

### Western blot analysis

Cells were treated with trametinib at the indicated concentrations and times, and then lysed in RIPA buffer containing protease inhibitors. Equal quantities (150 μg protein per lane) of total proteins were subjected to 10% SDS-PAGE, and then electrophoretically transferred onto PVDF membranes (Roche Diagnostics, Mannheim, Germany). The membranes were then incubated with the indicated primary antibodies at 4°C overnight. Anti-p-ERK1/2 and anti-total-ERK1 (t-ERK) were purchased from Bioworld Technology. Anti-GAPDH was purchased from Abmart. Anti-β-catenin was purchased from Cell Signaling Technology. This was followed by incubation with species-specific HRP-conjugated secondary antibodies from ZSGB-BIO, and antigen-antibody complexes were visualized using the Western Bright ECL detection system (Advansta, CA).

### Immunofluorescence assay

Cells were grown on sterile coverslips placed in 6-well plate, and subjected to treatment with trametinib at the indicated concentration for 48 h. Cells were then fixed with 4% paraformaldehyde for 15 min at room temperature, and permeabilized with 0.3% Triton X-100 for 10 min. After blocking with goat serum for 30 min, cells were incubated with primary antibody (Cell Signaling Technology, rabbit monoclonal antibody against β-catenin, 1:100 dilution) for overnight at 4°C. Cells were then incubated with goat-anti-rabbit secondary antibody for 90 min at room temperature in the dark. For nuclear counterstaining, cells were incubated with Hoechst 33342 for 5 min. Finally, cells were visualized using microscope, and images were acquired at 200× total magnification.

### Cell proliferation assay

Cell proliferation was measured by the MTT [3-(4, 5-Dimethylthiazolyl-2)-2, 5-diphenyltetrazolium bromide] assay. Briefly, GBC cells were cultured in 96-well plates. After trametinib treatment at the indicated times, 20 μl of 5 mg/ml MTT (Sigma, Saint Louis, MO) was added into the medium and further incubated for 4 h at 37°C, followed by addition of 150 μl of DMSO for 15 min. The plates were then read on a microplate reader using a test wavelength of 570 nm and a reference wavelength of 670 nm. Three triplicates were done to determine each data point.

### Colony formation assay

GBC cells in the logarithmic growth phase were digested into a single-cell suspension with a trypsin-EDTA solution, and 2 ml of cell suspension was then seeded onto 6-well plates at a density of 200 cells/ml. After adherence, cells were treated with the indicated concentration of trametinib. The medium was refreshed every 3 days. After 15 days of culture, surviving colonies (≥ 50 cells per colony) were fixed with methanol and stained with 0.5% crystal violet, and the colonies were then counted. Each experiment was performed in triplicate.

### Cell apoptosis assay

Cells (1 × 10^5^/well) were plated into 6-well plates, and treated with the indicated concentration of trametinib. After 48 h-incubation, the cells were collected and washed with cold PBS, centrifuged and subjected to sequential staining with Annexin V-FITC/ PI Apoptosis Detection Kit (Roche Applied Science). A total of at least 10000 events were collected and analyzed by two-color flow cytometer (BD Biosciences, NJ). Each experiment was performed in triplicate.

### Cell migration and invasion assays

The ability of cell migration and invasion was assessed by transwell chambers (8.0 μm pore size; Millipore, MA). For cell invasion assay, the chambers were pre-coated with 24 μg/μl of Matrigel (BD Bioscience, NJ) in 24-well plates. Cells (1 × 10^5^/well) were seeded in the upper chamber in 200 μl of medium containing 0.5% FBS and the indicated concentrations of trametinib or vehicle control. Medium with 20% FBS (1 ml) was added to the lower chamber. Following a 48 h-incubation at 37°C with 5% CO_2_, non-migrating and non-invading cells in the upper chamber were removed with a cotton swab, migrating and invading cells were fixed in 100% methanol and stained with 0.5% crystal violet in 2% ethanol. Photographs were taken randomly for at least five fields of each membrane using a microscope. The number of migrating or invading cells was expressed as the average number of cells per microscopic field over five fields. Each experiment was performed in triplicate.

### Tumor xenograft studies

Male athymic nude mice were purchased from SLAC laboratory Animal Co., Ltd. (Shanghai, PR. China) and housed in a specific pathogen-free (SPF) environment. NOZ cells (4 × 10^6^) were implanted in nude mice and grown as tumor xenografts. Dosing began when tumors achieved approximately 150 to 200 mm^3^. Trametinib (1 mg/kg) or vehicle was administered by oral gavage at a dose volume of 0.2 ml/20g body weight in 1.0% hydroxypropylmethylcellulose (Sigma). Dosing was daily for 9 consecutive days. Tumors were measured every other day, and tumor volume was estimated using the equation [Tumor volume (mm^3^) = (length×width^2^) × 0.5]. After treatment, tumors were harvested and weighted. Tumors tissues from every mice were fixed in 15% formalin for 24 h, and then embedded in paraffin, sectioned at 4 μm, and stained with hematoxylin and eosin (H&E). Cell proliferation ability was assessed by quantification with Ki-67 immunohistochemistry.

### Statistical analysis

Data were compared using the *t*-test (SPSS statistical package 16.0, Chicago, IL). *P* < 0.05 were considered significant. All values were expressed as the mean ± SD. Unless indicated, the data shown in the figures are representatives.

## SUPPLEMENTARY FIGURES AND TABLES






