# Supplementary material for: Identification of MAP kinase pathways as therapeutic targets in gallbladder carcinoma using targeted parallel sequencing
Source: Oncotarget. 2017 Mar 31;8(22):36319–30. doi: 10.18632/oncotarget.16751 (PMC5482657; doi:10.18632/oncotarget.16751)
Supplement: Supplementary file 1 [file oncotarget-08-36319-s001.pdf]

Identification of MAP kinase pathways as therapeutic targets in gallbladder carcinoma using targeted parallel sequencing

Supplementary Materials

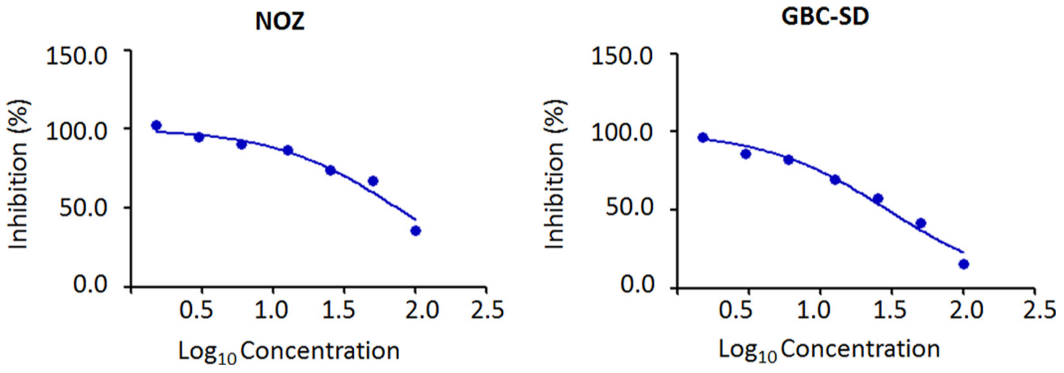

Figure 1: NOZ and GBC-SD cells were treated with different doses of iCRT-14 for 48 h. The MTT assay was performed to evaluate cell growth ability.

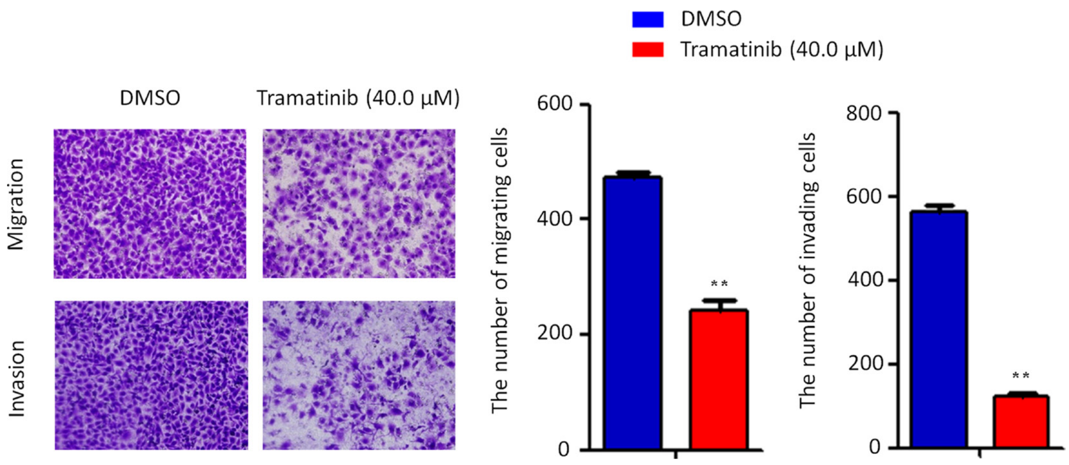

Figure 2: Trametinib inhibited cell migration and invasion in GBC-SD cells. The representative images of migrated/invaded cells (left panels). Histograms, corresponding to upper panels, show means ± SD of cell numbers from three independent assays (right panels). Statistically significant differences were indicated: $^{**}P < 0.01$.

Supplementary Table 1: Clinicopathologic characteristics of GBC patients used in this study for targeted parallel sequencing

Sample ID	Age at study entry	Gender	Diagnosis	TNM Stage	Tumor site	Histopathological subtypes	Presence of jaundice	Presence of gallbladder stones
T4	66	F	GBC	$T_3N_1M_0$	Fundus/body	low	NO	YES
T5	54	F	GBC	$T_3N_0M_0$	Fundus/body	low	NO	NO
T7	73	F	GBC	$T_3N_1M_0$	Fundus/body	low	NO	NO
T9	68	M	GBC	$T_3N_2M_0$	Fundus/body	low	NO	NO
T13	92	F	GBC	$T_3N_1M_0$	Neck	moderate	NO	NO
T14	48	F	GBC	$T_3N_1M_0$	Fundus/body	low	NO	YES
T15	61	M	GBC	$T_2N_1M_0$	Fundus/body	high	NO	NO
T16	79	M	GBC	$T_3N_1M_0$	Neck	low	NO	YES
T17	66	M	GBC	$T_3N_1M_0$	Fundus/body	high	NO	NO
T30	63	M	GBC	$T_4N_0M_0$	Fundus/body	low	YES	NO
T31	62	F	GBC	$T_3N_1M_1$	Fundus/body	moderate	YES	NO
T34	65	F	GBC	$T_3N_1M_0$	Fundus/body	moderate	NO	YES
T36	49	M	GBC	$T_4N_1M_1$	Fundus/body	low	NO	YES
T44	52	F	GBC	$T_3N_2M_0$	Fundus/body	low	NO	NO

Supplementary Table 2: A list of target genes. See Supplementary_Table_2

Supplementary Table 3: Recurrent mutations identified in this study. See Supplementary_Table_3
