# Supplementary material for: Identification of MAP kinase pathways as therapeutic targets in gallbladder carcinoma using targeted parallel sequencing
Source: Oncotarget. 2017 Mar 31;8(22):36319–30. doi: 10.18632/oncotarget.16751 (PMC5482657; doi:10.18632/oncotarget.16751)
Supplement: Supplementary file 2 [file oncotarget-08-36319-s002.docx]

**Supplementary Table 2:** **A list of target genes**

| **Symbol** | **Name** | **Gene ID** | **Chromosome** | **Chr Band** | **Cancer Somatic Mutations** | **Tumor Types** |
| --- | --- | --- | --- | --- | --- | --- |
| ABL1 | v-abl Abelson murine leukemia viral oncogene homolog 1 | 25 | 9 | 9q34.1 | yes | CML, ALL, T-ALL |
| ABL2 | v-abl Abelson murine leukemia viral oncogene homolog 2 | 27 | 1 | 1q24-q25 | yes | AML |
| AKT1 | v-akt murine thymoma viral oncogene homolog 1 | 207 | 14 | 14q32.32 | yes | breast, colorectal, ovarian, NSCLC |
| AKT2 | v-akt murine thymoma viral oncogene homolog 2 | 208 | 19 | 19q13.1-q13.2 | yes | ovarian, pancreatic |
| ALDH2 | aldehyde dehydrogenase 2 family (mitochondrial) | 217 | 12 | 12q24.2 | yes | leiomyoma |
| ALK | anaplastic lymphoma kinase (Ki-1) | 238 | 2 | 2p23 | yes | ALCL, NSCLC, neuroblastoma |
| APC | adenomatous polyposis of the colon gene | 324 | 5 | 5q21 | yes | colorectal, pancreatic, desmoid, hepatoblastoma, glioma, other CNS |
| BIRC3 | baculoviral IAP repeat-containing 3 | 330 | 11 | 11q22-q23 | yes | MALT |
| TNFRSF6 | tumor necrosis factor receptor superfamily, member 6 (FAS) | 355 | 10 | 10q24.1 | yes | TGCT, nasal NK/T lymphoma, skin squamous cell carcinoma-burn scar related |
| ARHH | RAS homolog gene family, member H (TTF) | 399 | 4 | 4p13 | yes | NHL |
| ARNT | aryl hydrocarbon receptor nuclear translocator | 405 | 1 | 1q21 | yes | AML |
| ATF1 | activating transcription factor 1 | 466 | 12 | 12q13 | yes | malignant melanoma of soft parts, angiomatoid fibrous histiocytoma |
| ATIC | 5-aminoimidazole-4-carboxamide ribonucleotide formyltransferase/IMP cyclohydrolase | 471 | 2 | 2q35 | yes | ALCL |
| ATM | ataxia telangiectasia mutated | 472 | 11 | 11q22.3 | yes | T-PLL |
| ATRX | alpha thalassemia/mental retardation syndrome X-linked | 546 | X | Xq21.1 | yes | Pancreatic neuroendocrine tumours, paediatric GBM |
| CCND1 | cyclin D1 | 595 | 11 | 11q13 | yes | CLL, B-ALL, breast |
| BCL2 | B-cell CLL/lymphoma 2 | 596 | 18 | 18q21.3 | yes | NHL, CLL |
| BCL3 | B-cell CLL/lymphoma 3 | 602 | 19 | 19q13 | yes | CLL |
| BCL5 | B-cell CLL/lymphoma 5 | 603 | 17 | 17q22 | yes | CLL |
| BCL6 | B-cell CLL/lymphoma 6 | 604 | 3 | 3q27 | yes | NHL, CLL |
| BCL7A | B-cell CLL/lymphoma 7A | 605 | 12 | 12q24.1 | yes | BNHL |
| BCL9 | B-cell CLL/lymphoma 9 | 607 | 1 | 1q21 | yes | B-ALL |
| TNFRSF17 | tumor necrosis factor receptor superfamily, member 17 | 608 | 16 | 16p13.1 | yes | intestinal T-cell lymphoma |
| BCR | breakpoint cluster region | 613 | 22 | 22q11.21 | yes | CML, ALL, AML |
| PRDM1 | PR domain containing 1, with ZNF domain | 639 | 6 | 6q21 | yes | DLBCL |
| BLM | Bloom Syndrome | 641 | 15 | 15q26.1 |  |  |
| BMPR1A | bone morphogenetic protein receptor, type IA | 657 | 10 | 10q22.3 |  |  |
| FOXL2 | forkhead box L2 | 668 | 3 | 3q23 | yes | granulosa-cell tumour of the ovary |
| BRCA1 | familial breast/ovarian cancer gene 1 | 672 | 17 | 17q21 | yes | ovarian |
| BRAF | v-raf murine sarcoma viral oncogene homolog B1 | 673 | 7 | 7q34 | yes | melanoma, colorectal, papillary thyroid, borderline ovarian, NSCLC, cholangiocarcinoma, pilocytic astrocytoma |
| BRCA2 | familial breast/ovarian cancer gene 2 | 675 | 13 | 13q12 | yes | breast, ovarian, pancreatic |
| BTG1 | B-cell translocation gene 1, anti-proliferative | 694 | 12 | 12q22 | yes | BCLL |
| BUB1B | BUB1 budding uninhibited by benzimidazoles 1 homolog beta (yeast) | 701 | 15 | 15q15 |  |  |
| CARS | cysteinyl-tRNA synthetase | 833 | 11 | 11p15.5 | yes | ALCL |
| RUNX1 | runt-related transcription factor 1 (AML1) | 861 | 21 | 21q22.3 | yes | AML, preB- ALL, T-ALL |
| CBFA2T1 | core-binding factor, runt domain, alpha subunit 2;translocated to, 1 (ETO) | 862 | 8 | 8q22 | yes | AML |
| CBFA2T3 | core-binding factor, runt domain, alpha subunit 2; translocated to, 3 (MTG-16) | 863 | 16 | 16q24 | yes | AML |
| CBFB | core-binding factor, beta subunit | 865 | 16 | 16q22 | yes | AML |
| CBL | Cas-Br-M (murine) ecotropic retroviral transforming | 867 | 11 | 11q23.3 | yes | AML, JMML, MDS |
| CBLB | Cas-Br-M (murine) ecotropic retroviral transforming sequence b | 868 | 3 | 3q13.11 | yes | AML |
| CCND2 | cyclin D2 | 894 | 12 | 12p13 | yes | NHL,CLL |
| CCND3 | cyclin D3 | 896 | 6 | 6p21 | yes | MM |
| CCNE1 | cyclin E1 | 898 | 19 | 19q12 | yes | serous ovarian |
| CD74 | CD74 molecule, major histocompatibility complex, class II invariant chain | 972 | 5 | 5q32 | yes | NSCLC |
| CD79A | CD79a molecule, immunoglobulin-associated alpha | 973 | 19 | 19q13.2 | yes | DLBCL |
| CD79B | CD79b molecule, immunoglobulin-associated beta | 974 | 17 | 17q23 | yes | DLBCL |
| CDH1 | cadherin 1, type 1, E-cadherin (epithelial) (ECAD) | 999 | 16 | 16q22.1 | yes | lobular breast, gastric |
| CDH11 | cadherin 11, type 2, OB-cadherin (osteoblast) | 1009 | 16 | 16q22.1 | yes | aneurysmal bone cyst |
| CDK4 | cyclin-dependent kinase 4 | 1019 | 12 | 12q14 |  |  |
| CDK6 | cyclin-dependent kinase 6 | 1021 | 7 | 7q21-q22 | yes | ALL |
| CDKN2A | cyclin-dependent kinase inhibitor 2A (p16(INK4a)) gene | 1029 | 9 | 9p21 | yes | melanoma, multiple other tumour types |
| CDKN2C | cyclin-dependent kinase inhibitor 2C (p18, inhibits CDK4) | 1031 | 1 | 1p32 | yes | glioma, MM |
| CDX2 | caudal type homeo box transcription factor 2 | 1045 | 13 | 13q12.3 | yes | AML |
| CEBPA | CCAAT/enhancer binding protein (C/EBP), alpha | 1050 | 19 | 19q13.1 | yes | AML, MDS |
| CHN1 | chimerin (chimaerin) 1 | 1123 | 2 | 2q31-q32.1 | yes | extraskeletal myxoid chondrosarcoma |
| CLTC | clathrin, heavy polypeptide (Hc) | 1213 | 17 | 17q11-qter | yes | ALCL, renal |
| COL1A1 | collagen, type I, alpha 1 | 1277 | 17 | 17q21.31-q22 | yes | dermatofibrosarcoma protuberans, aneurysmal bone cyst |
| COPEB | core promoter element binding protein (KLF6) | 1316 | 10 | 10p15 | yes | prostate, glioma |
| COX6C | cytochrome c oxidase subunit VIc | 1345 | 8 | 8q22-q23 | yes | uterine leiomyoma |
| CREB1 | cAMP responsive element binding protein 1 | 1385 | 2 | 2q34 | yes | clear cell sarcoma, angiomatoid fibrous histiocytoma |
| CREBBP | CREB binding protein (CBP) | 1387 | 16 | 16p13.3 | yes | ALL, AML, DLBCL, B-NHL |
| CTNNB1 | catenin (cadherin-associated protein), beta 1 | 1499 | 3 | 3p22-p21.3 | yes | colorectal, ovarian, hepatoblastoma, pleomorphic salivary gland adenoma, other tumour types |
| CYLD | familial cylindromatosis gene | 1540 | 16 | 16q12-q13 | yes | cylindroma |
| DAXX | death-domain associated protein | 1616 | 6 | 6p21.3 | yes | pancreatic neuroendocrine tumour, paediatric glioblastoma |
| DDB2 | damage-specific DNA binding protein 2 | 1643 | 11 | 11p12 |  |  |
| DDIT3 | DNA-damage-inducible transcript 3 | 1649 | 12 | 12q13.1-q13.2 | yes | liposarcoma |
| DDX5 | DEAD (Asp-Glu-Ala-Asp) box polypeptide 5 | 1655 | 17 | 17q21 | yes | prostate |
| DDX6 | DEAD (Asp-Glu-Ala-Asp) box polypeptide 6 | 1656 | 11 | 11q23.3 | yes | B-NHL |
| DDX10 | DEAD (Asp-Glu-Ala-Asp) box polypeptide 10 | 1662 | 11 | 11q22-q23 | yes | AML* |
| DNM2 | dynamin 2 | 1785 | 19 | 19p13.2 | yes | ETP ALL |
| DNMT3A | DNA (cytosine-5-)-methyltransferase 3 alpha | 1788 | 2 | 2p23 | yes | AML |
| EBF1 | early B-cell factor 1 | 1879 | 5 | 5q34 | yes | lipoma |
| EGFR | epidermal growth factor receptor (erythroblastic leukemia viral (v-erb-b) oncogene homolog, avian) | 1956 | 7 | 7p12.3-p12.1 | yes | glioma, NSCLC |
| EIF4A2 | eukaryotic translation initiation factor 4A, isoform 2 | 1974 | 3 | 3q27.3 | yes | NHL |
| ELF4 | E74-like factor 4 (ets domain transcription factor) | 2000 | X | Xq26 | yes | AML |
| ELK4 | ELK4, ETS-domain protein (SRF accessory protein 1) | 2005 | 1 | 1q32 | yes | prostate |
| ELN | elastin | 2006 | 7 | 7q11.23 | yes | B-ALL |
| EP300 | 300 kd E1A-Binding protein gene | 2033 | 22 | 22q13 | yes | colorectal, breast, pancreatic, AML, ALL, DLBCL |
| EPS15 | epidermal growth factor receptor pathway substrate 15 (AF1p) | 2060 | 1 | 1p32 | yes | ALL |
| ERBB2 | v-erb-b2 erythroblastic leukemia viral oncogene homolog 2, neuro/glioblastoma derived oncogene homolog (avian) | 2064 | 17 | 17q21.1 | yes | breast, ovarian, other tumour types, NSCLC, gastric |
| ERCC2 | excision repair cross-complementing rodent repair deficiency, complementation group 2 (xeroderma pigmentosum D) | 2068 | 19 | 19q13.2-q13.3 |  |  |
| ERCC3 | excision repair cross-complementing rodent repair deficiency, complementation group 3 (xeroderma pigmentosum group B complementing) | 2071 | 2 | 2q21 |  |  |
| ERCC4 | excision repair cross-complementing rodent repair deficiency, complementation group 4 | 2072 | 16 | 16p13.3-p13.13 |  |  |
| ERCC5 | excision repair cross-complementing rodent repair deficiency, complementation group 5 (xeroderma pigmentosum, complementation group G (Cockayne syndrome)) | 2073 | 13 | 13q33 |  |  |
| ERG | v-ets erythroblastosis virus E26 oncogene like (avian) | 2078 | 21 | 21q22.3 | yes | Ewing sarcoma, prostate, AML |
| ETV1 | ets variant gene 1 | 2115 | 7 | 7p22 | yes | Ewing sarcoma, prostate |
| ETV4 | ets variant gene 4 (E1A enhancer binding protein, E1AF) | 2118 | 17 | 17q21 | yes | Ewing sarcoma, prostate carcinoma |
| ETV5 | ets variant gene 5 | 2119 | 3 | 3q28 | yes | prostate |
| ETV6 | ets variant gene 6 (TEL oncogene) | 2120 | 12 | 12p13 | yes | congenital fibrosarcoma, multiple leukaemia and lymphoma, secretory breast, MDS, ALL |
| EVI1 | ecotropic viral integration site 1 | 2122 | 3 | 3q26 | yes | AML, CML |
| EWSR1 | Ewing sarcoma breakpoint region 1 (EWS) | 2130 | 22 | 22q12 | yes | Ewing sarcoma, desmoplastic small round cell tumour , ALL, clear cell sarcoma, sarcoma, myoepithelioma |
| EXT1 | multiple exostoses type 1 gene | 2131 | 8 | 8q24.11-q24.13 |  |  |
| EXT2 | multiple exostoses type 2 gene | 2132 | 11 | 11p12-p11 |  |  |
| EZH2 | enhancer of zeste homolog 2 | 2146 | 7 | 7q35-q36 | yes | DLBCL |
| FANCA | Fanconi anemia, complementation group A | 2175 | 16 | 16q24.3 |  |  |
| FANCC | Fanconi anemia, complementation group C | 2176 | 9 | 9q22.3 |  |  |
| FANCD2 | Fanconi anemia, complementation group D2 | 2177 | 3 | 3p26 |  |  |
| FANCE | Fanconi anemia, complementation group E | 2178 | 6 | 6p21-p22 |  |  |
| ACSL3 | acyl-CoA synthetase long-chain family member 3 | 2181 | 2 | 2q36 | yes | prostate |
| FANCF | Fanconi anemia, complementation group F | 2188 | 11 | 11p15 |  |  |
| FANCG | Fanconi anemia, complementation group G | 2189 | 9 | 9p13 |  |  |
| FCGR2B | Fc fragment of IgG, low affinity IIb, receptor for (CD32) | 2213 | 1 | 1q23 | yes | ALL |
| FGFR1 | fibroblast growth factor receptor 1 | 2260 | 8 | 8p11.2-p11.1 | yes | MPD, NHL |
| FGFR3 | fibroblast growth factor receptor 3 | 2261 | 4 | 4p16.3 | yes | bladder, MM, T-cell lymphoma |
| FGFR2 | fibroblast growth factor receptor 2 | 2263 | 10 | 10q26 | yes | gastric, NSCLC, endometrial |
| FH | fumarate hydratase | 2271 | 1 | 1q42.1 |  |  |
| FHIT | fragile histidine triad gene | 2272 | 3 | 3p14.2 | yes | pleomorphic salivary gland adenoma |
| FOXO1A | forkhead box O1A (FKHR) | 2308 | 13 | 13q14.1 | yes | alveolar rhabdomyosarcoma |
| FOXO3A | forkhead box O3A | 2309 | 6 | 6q21 | yes | AL |
| FLI1 | Friend leukemia virus integration 1 | 2313 | 11 | 11q24 | yes | Ewing sarcoma |
| FLT3 | fms-related tyrosine kinase 3 | 2322 | 13 | 13q12 | yes | AML, ALL |
| FUS | fusion, derived from t(12;16) malignant liposarcoma | 2521 | 16 | 16p11.2 | yes | liposarcoma, AML, Ewing sarcoma, angiomatoid fibrous histiocytoma, fibromyxoid sarcoma |
| FVT1 | follicular lymphoma variant translocation 1 | 2531 | 18 | 18q21.3 | yes | B-NHL |
| GATA1 | GATA binding protein 1 (globin transcription factor 1) | 2623 | X | Xp11.23 | yes | megakaryoblastic leukaemia of Downs syndrome |
| GATA2 | GATA binding protein 2 | 2624 | 3 | 3q21.3 | yes | AML (CML blast transformation) |
| GATA3 | GATA binding protein 3 | 2625 | 10 | 10p15 | yes | breast |
| GPC3 | glypican 3 | 2719 | X | Xq26.1 |  |  |
| GNA11 | guanine nucleotide binding protein (G protein), alpha 11 (Gq class) | 2767 | 19 | 19p13.3 | yes | uveal melanoma |
| GNAQ | guanine nucleotide binding protein (G protein), q polypeptide | 2776 | 9 | 9q21 | yes | uveal melanoma |
| GNAS | guanine nucleotide binding protein (G protein), alpha stimulating activity polypeptide 1 | 2778 | 20 | 20q13.2 | yes | pituitary adenoma |
| MSH6 | mutS homolog 6 (E. coli) | 2956 | 2 | 2p16 | yes | colorectal |
| H3F3A | H3 histone, family 3A | 3020 | 1 | 1q42.12 | yes | glioma |
| HIP1 | huntingtin interacting protein 1 | 3092 | 7 | 7q11.23 | yes | CMML |
| HLXB9 | homeo box HB9 | 3110 | 7 | 7q36 | yes | AML |
| HLF | hepatic leukemia factor | 3131 | 17 | 17q22 | yes | ALL |
| HMGA1 | high mobility group AT-hook 1 | 3159 | 6 | 6p21 | yes | microfollicular thyroid adenoma, various benign mesenchymal tumours |
| HNRNPA2B1 | heterogeneous nuclear ribonucleoprotein A2/B1 | 3181 | 7 | 7p15 | yes | prostate |
| TLX1 | T-cell leukemia, homeobox 1 (HOX11) | 3195 | 10 | 10q24 | yes | T-ALL |
| HOXA9 | homeo box A9 | 3205 | 7 | 7p15-p14.2 | yes | AML* |
| HOXA11 | homeo box A11 | 3207 | 7 | 7p15-p14.2 | yes | CML |
| HOXA13 | homeo box A13 | 3209 | 7 | 7p15-p14.2 | yes | AML |
| HOXC11 | homeo box C11 | 3227 | 12 | 12q13.3 | yes | AML |
| HOXC13 | homeo box C13 | 3229 | 12 | 12q13.3 | yes | AML |
| HOXD11 | homeo box D11 | 3237 | 2 | 2q31-q32 | yes | AML |
| HOXD13 | homeo box D13 | 3239 | 2 | 2q31-q32 | yes | AML* |
| HRAS | v-Ha-ras Harvey rat sarcoma viral oncogene homolog | 3265 | 11 | 11p15.5 | yes | infrequent sarcomas, rare other tumour types |
| HRPT2 | hyperparathyroidism 2 | 3279 | 1 | 1q21-q31 | yes | parathyroid adenoma |
| HSPCA | heat shock 90kDa protein 1, alpha | 3320 | 14 | 14q32.31 | yes | NHL |
| HSPCB | heat shock 90kDa protein 1, beta | 3326 | 6 | 6p12 | yes | NHL |
| IDH1 | isocitrate dehydrogenase 1 (NADP+), soluble | 3417 | 2 | 2q33.3 | yes | glioblastoma |
| IDH2 | socitrate dehydrogenase 2 (NADP+), mitochondrial | 3418 | 15 | 15q26.1 | yes | glioblastoma |
| IGH@ | immunoglobulin heavy locus | 3492 | 14 | 14q32.33 | yes | MM, Burkitt lymphoma, NHL, CLL, B-ALL, MALT, MLCLS |
| IGL@ | immunoglobulin lambda locus | 3535 | 22 | 22q11.1-q11.2 | yes | Burkitt lymphoma |
| IL2 | interleukin 2 | 3558 | 4 | 4q26-q27 | yes | intestinal T-cell lymphoma |
| IL6ST | interleukin 6 signal transducer (gp130, oncostatin M receptor) | 3572 | 5 | 5q11 | yes | hepatocellular carcinoma |
| IL7R | interleukin 7 receptor | 3575 | 5 | 5p13 | yes | ALL, ETP ALL |
| IRF4 | interferon regulatory factor 4 | 3662 | 6 | 6p25-p23 | yes | MM |
| ITK | IL2-inducible T-cell kinase | 3702 | 5 | 5q31-q32 | yes | peripheral T-cell lymphoma |
| JAK1 | Janus kinase 1 | 3716 | 1 | 1p32.3-p31.3 | yes | ALL |
| JAK2 | Janus kinase 2 | 3717 | 9 | 9p24 | yes | ALL, AML, MPD, CML |
| JAK3 | Janus kinase 3 | 3718 | 19 | 19p13.1 | yes | acute megakaryocytic leukaemia, ETP ALL |
| JUN | jun oncogene | 3725 | 1 | 1p32-p31 | yes | sarcoma |
| KCNJ5 | potassium inwardly-rectifying channel, subfamily J, member 5 | 3762 | 11 | 11q24 | yes | adrenal |
| KDR | vascular endothelial growth factor receptor 2 | 3791 | 4 | 4q11-q12 | yes | NSCLC, angiosarcoma |
| KIF5B | kinesin family member 5B | 3799 | 10 | 10p11.22 | yes | NSCLC |
| KIT | v-kit Hardy-Zuckerman 4 feline sarcoma viral oncogene homolog | 3815 | 4 | 4q12 | yes | GIST, AML, TGCT, mastocytosis, mucosal melanoma |
| KLK2 | kallikrein-related peptidase 2 | 3817 | 19 | 19q13.41 | yes | prostate |
| KRAS | v-Ki-ras2 Kirsten rat sarcoma 2 viral oncogene homolog | 3845 | 12 | 12p12.1 | yes | pancreatic, colorectal, lung, thyroid, AML, other tumour types |
| KTN1 | kinectin 1 (kinesin receptor) | 3895 | 14 | 14q22.1 | yes | papillary thyroid |
| LAF4 | lymphoid nuclear protein related to AF4 | 3899 | 2 | 2q11.2-q12 | yes | ALL, T-ALL |
| LASP1 | LIM and SH3 protein 1 | 3927 | 17 | 17q11-q21.3 | yes | AML |
| LCK | lymphocyte-specific protein tyrosine kinase | 3932 | 1 | 1p35-p34.3 | yes | T-ALL |
| LCP1 | lymphocyte cytosolic protein 1 (L-plastin) | 3936 | 13 | 13q14.1-q14.3 | yes | NHL |
| LIFR | leukemia inhibitory factor receptor | 3977 | 5 | 5p13-p12 | yes | salivary adenoma |
| LMO1 | LIM domain only 1 (rhombotin 1) (RBTN1) | 4004 | 11 | 11p15 | yes | T-ALL, neuroblastoma |
| LMO2 | LIM domain only 2 (rhombotin-like 1) (RBTN2) | 4005 | 11 | 11p13 | yes | T-ALL |
| LPP | LIM domain containing preferred translocation partner in lipoma | 4026 | 3 | 3q28 | yes | lipoma, leukaemia |
| LYL1 | lymphoblastic leukemia derived sequence 1 | 4066 | 19 | 19p13.2-p13.1 | yes | T-ALL |
| MADH4 | Homolog of Drosophila Mothers Against Decapentaplegic 4 gene | 4089 | 18 | 18q21.1 | yes | colorectal, pancreatic, small intestine |
| MAF | v-maf musculoaponeurotic fibrosarcoma oncogene homolog | 4094 | 16 | 16q22-q23 | yes | MM |
| MAX | Myc associated factor X | 4149 | 14 | 14q23 | yes | pheochromocytoma |
| MDM2 | Mdm2 p53 binding protein homolog | 4193 | 12 | 12q15 | yes | sarcoma, glioma, colorectal, other tumour types |
| MDM4 | Mdm4 p53 binding protein homolog | 4194 | 1 | 1q32 | yes | glioblastoma, bladder, retinoblastoma |
| MEN1 | multiple endocrine neoplasia type 1 gene | 4221 | 11 | 11q13 | yes | parathyroid tumours, pancreatic neuroendocrine tumour |
| MET | met proto-oncogene (hepatocyte growth factor receptor) | 4233 | 7 | 7q31 | yes | papillary renal, head-neck squamous cell |
| CIITA | class II, major histocompatibility complex, transactivator | 4261 | 16 | 16p13 | yes | PMBL, Hodgkin lymphoma |
| MITF | microphthalmia-associated transcription factor | 4286 | 3 | 3p14.1 | yes | melanoma |
| MLF1 | myeloid leukemia factor 1 | 4291 | 3 | 3q25.1 | yes | AML |
| MLH1 | E.coli MutL homolog gene | 4292 | 3 | 3p21.3 | yes | colorectal, endometrial, ovarian, CNS |
| MLL | myeloid/lymphoid or mixed-lineage leukemia (trithorax homolog, Drosophila) | 4297 | 11 | 11q23 | yes | AML, ALL |
| MLLT1 | myeloid/lymphoid or mixed-lineage leukemia (trithorax homolog, Drosophila); translocated to, 1 (ENL) | 4298 | 19 | 19p13.3 | yes | AL |
| MLLT2 | myeloid/lymphoid or mixed-lineage leukemia (trithorax homolog, Drosophila); translocated to, 2 (AF4) | 4299 | 4 | 4q21 | yes | AL |
| MLLT3 | myeloid/lymphoid or mixed-lineage leukemia (trithorax homolog, Drosophila); translocated to, 3 (AF9) | 4300 | 9 | 9p22 | yes | ALL |
| MLLT4 | myeloid/lymphoid or mixed-lineage leukemia (trithorax homolog, Drosophila); translocated to, 4 (AF6) | 4301 | 6 | 6q27 | yes | AL |
| MLLT6 | myeloid/lymphoid or mixed-lineage leukemia (trithorax homolog, Drosophila); translocated to, 6 (AF17) | 4302 | 17 | 17q21 | yes | AL |
| MLLT7 | myeloid/lymphoid or mixed-lineage leukemia (trithorax homolog, Drosophila); translocated to, 7 (AFX1) | 4303 | X | Xq13.1 | yes | AL |
| MN1 | meningioma (disrupted in balanced translocation) 1 | 4330 | 22 | 22q13 | yes | AML, meningioma |
| MPL | myeloproliferative leukemia virus oncogene, thrombopoietin receptor | 4352 | 1 | p34 | yes | MPD |
| MSH2 | mutS homolog 2 (E. coli) | 4436 | 2 | 2p22-p21 | yes | colorectal, endometrial, ovarian |
| MSN | moesin | 4478 | X | Xq11.2-q12 | yes | ALCL |
| MTCP1 | mature T-cell proliferation 1 | 4515 | X | Xq28 | yes | T cell prolymphocytic leukaemia |
| MUC1 | mucin 1, transmembrane | 4582 | 1 | 1q21 | yes | B-NHL |
| MUTYH | mutY homolog (E. coli) | 4595 | 1 | 1p34.3-1p32.1 |  |  |
| MYB | v-myb myeloblastosis viral oncogene homolog | 4602 | 6 | 6q22-23 | yes | adenoid cystic carcinoma |
| MYC | v-myc myelocytomatosis viral oncogene homolog (avian) | 4609 | 8 | 8q24.12-q24.13 | yes | Burkitt lymphoma, amplified in other cancers, B-CLL |
| MYCL1 | v-myc myelocytomatosis viral oncogene homolog 1, lung carcinoma derived (avian) | 4610 | 1 | 1p34.3 | yes | small cell lung carcinoma |
| MYCN | v-myc myelocytomatosis viral related oncogene, neuroblastoma derived (avian) | 4613 | 2 | 2p24.1 | yes | neuroblastoma |
| MYD88 | myeloid differentiation primary response gene (88) | 4615 | 3 | 3p22 | yes | ABC-DLBCL |
| MYH9 | myosin, heavy polypeptide 9, non-muscle | 4627 | 22 | 22q13.1 | yes | ALCL |
| MYH11 | myosin, heavy polypeptide 11, smooth muscle | 4629 | 16 | 16p13.13-p13.12 | yes | AML |
| NACA | nascent-polypeptide-associated complex alpha polypeptide | 4666 | 12 | 12q23-q24.1 | yes | NHL |
| NBS1 | Nijmegen breakage syndrome 1 (nibrin) | 4683 | 8 | 8q21 |  |  |
| NF1 | neurofibromatosis type 1 gene | 4763 | 17 | 17q12 | yes | neurofibroma, glioma |
| NF2 | neurofibromatosis type 2 gene | 4771 | 22 | 22q12.2 | yes | meningioma, acoustic neuroma, renal |
| NFE2L2 | nuclear factor (erythroid-derived 2)-like 2 (NRF2) | 4780 | 2 | 2q31 | yes | NSCLC, HNSCC |
| NFIB | nuclear factor I/B | 4781 | 9 | 9p24.1 | yes | adenoid cystic carcinoma, lipoma |
| NFKB2 | nuclear factor of kappa light polypeptide gene enhancer in B-cells 2 (p49/p100) | 4791 | 10 | 10q24 | yes | B-NHL |
| NONO | non-POU domain containing, octamer-binding | 4841 | X | Xq13.1 | yes | papillary renal |
| CNOT3 | CCR4-NOT transcription complex subunit 3 | 4849 | 19 | 19q13.4 | yes | T-ALL |
| NOTCH1 | Notch homolog 1, translocation-associated (Drosophila) (TAN1) | 4851 | 9 | 9q34.3 | yes | T-ALL |
| NOTCH2 | Notch homolog 2 | 4853 | 1 | 1p13-p11 | yes | marginal zone lymphoma, DLBCL |
| NPM1 | nucleophosmin (nucleolar phosphoprotein B23, numatrin) | 4869 | 5 | 5q35 | yes | NHL, APL, AML |
| NRAS | neuroblastoma RAS viral (v-ras) oncogene homolog | 4893 | 1 | 1p13.2 | yes | melanoma, MM, AML, thyroid |
| NTRK1 | neurotrophic tyrosine kinase, receptor, type 1 | 4914 | 1 | 1q21-q22 | yes | papillary thyroid |
| NTRK3 | neurotrophic tyrosine kinase, receptor, type 3 | 4916 | 15 | 15q25 | yes | congenital fibrosarcoma, secretory breast |
| NUMA1 | nuclear mitotic apparatus protein 1 | 4926 | 11 | 11q13 | yes | APL |
| NUP98 | nucleoporin 98kDa | 4928 | 11 | 11p15 | yes | AML |
| OMD | osteomodulin | 4958 | 9 | 9q22.31 | yes | aneurysmal bone cyst |
| PAFAH1B2 | platelet-activating factor acetylhydrolase, isoform Ib, beta subunit 30kDa | 5049 | 11 | 11q23 | yes | MLCLS |
| PAX3 | paired box gene 3 | 5077 | 2 | 2q35 | yes | alveolar rhabdomyosarcoma |
| PAX5 | paired box gene 5 (B-cell lineage specific activator protein) | 5079 | 9 | 9p13 | yes | NHL, ALL, B-ALL |
| PAX7 | paired box gene 7 | 5081 | 1 | 1p36.2-p36.12 | yes | alveolar rhabdomyosarcoma |
| PBX1 | pre-B-cell leukemia transcription factor 1 | 5087 | 1 | 1q23 | yes | pre B-ALL, myoepithelioma |
| PCM1 | pericentriolar material 1 (PTC4) | 5108 | 8 | 8p22-p21.3 | yes | papillary thyroid, CML, MPD |
| PDGFB | platelet-derived growth factor beta polypeptide (simian sarcoma viral (v-sis) oncogene homolog) | 5155 | 22 | 22q12.3-q13.1 | yes | DFSP |
| PDGFRA | platelet-derived growth factor, alpha-receptor | 5156 | 4 | 4q11-q13 | yes | GIST, idiopathic hypereosinophilic syndrome, paediatric glioblastoma |
| PDGFRB | platelet-derived growth factor receptor, beta polypeptide | 5159 | 5 | 5q31-q32 | yes | MPD, AML, CMML, CML |
| PER1 | period homolog 1 (Drosophila) | 5187 | 17 | 17p13.1-17p12 | yes | AML, CMML |
| PIK3CA | phosphoinositide-3-kinase, catalytic, alpha polypeptide | 5290 | 3 | 3q26.3 | yes | colorectal, gastric, glioblastoma, breast |
| PIM1 | pim-1 oncogene | 5292 | 6 | 6p21.2 | yes | NHL |
| PIK3R1 | phosphoinositide-3-kinase, regulatory subunit 1 (alpha) | 5295 | 5 | 5q13.1 | yes | glioblastoma, ovarian, colorectal |
| PLAG1 | pleiomorphic adenoma gene 1 | 5324 | 8 | 8q12 | yes | salivary adenoma |
| PML | promyelocytic leukemia | 5371 | 15 | 15q22 | yes | APL, ALL |
| PMS1 | PMS1 postmeiotic segregation increased 1 (S. cerevisiae) | 5378 | 2 | 2q31-q33 |  |  |
| PMS2 | PMS2 postmeiotic segregation increased 2 (S. cerevisiae) | 5395 | 7 | 7p22 |  |  |
| PMX1 | paired mesoderm homeo box 1 | 5396 | 1 | 1q24 | yes | AML |
| PNUTL1 | peanut-like 1 (Drosophila) | 5413 | 22 | 22q11.2 | yes | AML |
| POU2AF1 | POU domain, class 2, associating factor 1 (OBF1) | 5450 | 11 | 11q23.1 | yes | NHL |
| POU5F1 | POU domain, class 5, transcription factor 1 | 5460 | 6 | 6p21.31 | yes | sarcoma |
| PPARG | peroxisome proliferative activated receptor, gamma | 5468 | 3 | 3p25 | yes | follicular thyroid |
| PPP2R1A | protein phosphatase 2, regulatory subunit A, alpha | 5518 | 19 | 19q13.41 | yes | clear cell ovarian carcinoma |
| PRCC | papillary renal cell carcinoma (translocation-associated) | 5546 | 1 | 1q21.1 | yes | papillary renal |
| PRF1 | perforin 1 (pore forming protein) | 5551 | 10 | 10q22 |  |  |
| PRKAR1A | protein kinase, cAMP-dependent, regulatory, type I, alpha (tissue specific extinguisher 1) | 5573 | 17 | 17q23-q24 | yes | papillary thyroid |
| MAP2K1 | mitogen-activated protein kinase kinase 1 | 5604 | 15 | 15q22.1-q22.33 | yes | NSCLC, melanoma, colorectal |
| MAP2K2 | mitogen-activated protein kinase kinase 2 | 5605 | 19 | 19p13.3 | yes | NSCLC, melanoma |
| PTCH | Homolog of Drosophila Patched gene | 5727 | 9 | 9q22.3 | yes | skin basal cell, medulloblastoma |
| PTEN | phosphatase and tensin homolog gene | 5728 | 10 | 10q23.3 | yes | glioma, prostate, endometrial |
| PTPN11 | protein tyrosine phosphatase, non-receptor type 11 | 5781 | 12 | 12q24.1 | yes | JMML, AML, MDS |
| RAC1 | ras-related C3 botulinum toxin substrate 1 (rho family, small GTP binding protein Rac1) | 5879 | 7 | 7p22 | yes | melanoma |
| RAD51L1 | RAD51-like 1 (S. cerevisiae) (RAD51B) | 5890 | 14 | 14q23-q24.2 | yes | lipoma, uterine leiomyoma |
| RAF1 | v-raf-1 murine leukemia viral oncogene homolog 1 | 5894 | 3 | 3p25 | yes | pilocytic astrocytoma |
| RALGDS | ral guanine nucleotide dissociation stimulator | 5900 | 9 | 9q34.3 | yes | PMBL, Hodgkin lymphoma, |
| RAP1GDS1 | RAP1, GTP-GDP dissociation stimulator 1 | 5910 | 4 | 4q21-q25 | yes | T-ALL |
| RARA | retinoic acid receptor, alpha | 5914 | 17 | 17q12 | yes | APL |
| RB1 | retinoblastoma gene | 5925 | 13 | 13q14 | yes | retinoblastoma, sarcoma, breast, small cell lung carcinoma |
| KDM5A | lysine (K)-specific demethylase 5A, JARID1A | 5927 | 12 | 12p11 | yes | AML |
| REL | v-rel reticuloendotheliosis viral oncogene homolog (avian) | 5966 | 2 | 2p13-p12 | yes | Hodgkin lymphoma |
| RET | ret proto-oncogene | 5979 | 10 | 10q11.2 | yes | medullary thyroid, papillary thyroid, pheochromocytoma, NSCLC |
| TRIM27 | tripartite motif-containing 27 | 5987 | 6 | 6p22 | yes | papillary thyroid |
| ROS1 | v-ros UR2 sarcoma virus oncogene homolog 1 (avian) | 6098 | 6 | 6q22 | yes | glioblastoma, NSCLC |
| RPL5 | ribososomal protein L5 | 6125 | 1 | 1p22.1 | yes | T-ALL |
| RPL10 | ribosomal protein L10 | 6134 | X | Xq28 | yes | T-ALL |
| RPL22 | ribosomal protein L22 (EAP) | 6146 | 1 | 1p36.31 | yes | AML, CML |
| RPN1 | ribophorin I | 6184 | 3 | 3q21.3-q25.2 | yes | AML |
| SDC4 | syndecan 4 | 6385 | 20 | 20q12 | yes | NSCLC |
| SDHB | succinate dehydrogenase complex, subunit B, iron sulfur (Ip) | 6390 | 1 | 1p36.1-p35 |  |  |
| SDHC | succinate dehydrogenase complex, subunit C, integral membrane protein, 15kDa | 6391 | 1 | 1q21 |  |  |
| SDHD | succinate dehydrogenase complex, subunit D, integral membrane protein | 6392 | 11 | 11q23 |  |  |
| MAP2K4 | mitogen-activated protein kinase kinase 4 | 6416 | 17 | 17p11.2 | yes | pancreatic, breast, colorectal |
| SET | SET translocation | 6418 | 9 | 9q34 | yes | AML |
| SFPQ | splicing factor proline/glutamine rich(polypyrimidine tract binding protein associated) | 6421 | 1 | 1p34.3 | yes | papillary renal |
| SRSF2 | serine/arginine-rich splicing factor 2 | 6427 | 17 | 17q25 | yes | MDS, CLL |
| SFRS3 | splicing factor, arginine/serine-rich 3 | 6428 | 6 | 6p21 | yes | follicular lymphoma |
| SH3GL1 | SH3-domain GRB2-like 1 (EEN) | 6455 | 19 | 19p13.3 | yes | AL |
| SIL | TAL1 (SCL) interrupting locus | 6491 | 1 | 1p32 | yes | T-ALL |
| SMARCA4 | SWI/SNF related, matrix associated, actin dependent regulator of chromatin, subfamily a, member 4 | 6597 | 19 | 19p13.2 | yes | NSCLC |
| SMARCB1 | SWI/SNF related, matrix associated, actin dependent regulator of chromatin, subfamily b, member 1 | 6598 | 22 | 22q11 | yes | malignant rhabdoid |
| SMARCE1 | SWI/SNF related, matrix associated, actin dependent regulator of chromatin, subfamily e, member 1 | 6605 | 17 | 17q21.2 |  |  |
| SMO | smoothened homolog (Drosophila) | 6608 | 7 | 7q31-q32 | yes | skin basal cell |
| SOX2 | SRY (sex determining region Y)-box 2 | 6657 | 3 | 3q26.3-q27 | yes | NSCLC, oesophageal squamous carcinoma |
| SSX1 | synovial sarcoma, X breakpoint 1 | 6756 | X | Xp11.23-p11.22 | yes | synovial sarcoma |
| SSX2 | synovial sarcoma, X breakpoint 2 | 6757 | X | Xp11.23-p11.22 | yes | synovial sarcoma |
| SSX4 | synovial sarcoma, X breakpoint 4 | 6759 | X | Xp11.23 | yes | synovial sarcoma |
| SS18 | synovial sarcoma translocation, chromosome 18 | 6760 | 18 | 18q11.2 | yes | synovial sarcoma |
| STAT3 | signal transducer and activator of transcription 3 (acute-phase response factor) | 6774 | 17 | 17q21.31 | yes | T-cell large granular lymphocytic lymphoma |
| STK11 | serine/threonine kinase 11 gene (LKB1) | 6794 | 19 | 19p13.3 | yes | NSCLC, pancreatic |
| SYK | spleen tyrosine kinase | 6850 | 9 | 9q22 | yes | MDS, peripheral T-cell lymphoma |
| TAL1 | T-cell acute lymphocytic leukemia 1 (SCL) | 6886 | 1 | 1p32 | yes | lymphoblastic leukaemia/biphasic |
| TAL2 | T-cell acute lymphocytic leukemia 2 | 6887 | 9 | 9q31 | yes | T-ALL |
| TCEA1 | transcription elongation factor A (SII), 1 | 6917 | 8 | 8q11.2 | yes | salivary adenoma |
| TCF1 | transcription factor 1, hepatic (HNF1) | 6927 | 12 | 12q24.2 | yes | hepatic adenoma, hepatocellular |
| TCF3 | transcription factor 3 (E2A immunoglobulin enhancer binding factors E12/E47) | 6929 | 19 | 19p13.3 | yes | pre B-ALL |
| TCF7L2 | transcription factor 7-like 2 | 6934 | 10 | 10q25.3 | yes | colorectal |
| TCF12 | transcription factor 12 (HTF4, helix-loop-helix transcription factors 4) | 6938 | 15 | 15q21 | yes | extraskeletal myxoid chondrosarcoma |
| TRA@ | T cell receptor alpha locus | 6955 | 14 | 14q11.2 | yes | T-ALL |
| TRB@ | T cell receptor beta locus | 6957 | 7 | 7q35 | yes | T-ALL |
| TRD@ | T cell receptor delta locus | 6964 | 14 | 14q11 | yes | T-cell leukaemia |
| TERT | telomerase reverse transcriptase | 7015 | 5 | 5p15.33 | yes | melanoma |
| TFE3 | transcription factor binding to IGHM enhancer 3 | 7030 | X | Xp11.22 | yes | papillary renal, alveolar soft part sarcoma, renal |
| TFRC | transferrin receptor (p90, CD71) | 7037 | 3 | 3q29 | yes | NHL |
| NKX2-1 | NK2 homeobox 1 | 7080 | 14 | 14q13 | yes | NSCLC |
| TMPRSS2 | transmembrane protease, serine 2 | 7113 | 21 | 21q22.3 | yes | prostate |
| TNFAIP3 | tumor necrosis factor, alpha-induced protein 3 | 7128 | 6 | 6q23 | yes | marginal zone B-cell lymphomas, Hodgkin lymphoma, PMBL |
| TOP1 | topoisomerase (DNA) I | 7150 | 20 | 20q12-q13.1 | yes | AML* |
| TP53 | tumor protein p53 | 7157 | 17 | 17p13 | yes | breast, colorectal, lung, sarcoma, adrenocortical, glioma, multiple other tumour types |
| TPM3 | tropomyosin 3 | 7170 | 1 | 1q22-q23 | yes | papillary thyroid, ALCL, NSCLC |
| TPM4 | tropomyosin 4 | 7171 | 19 | 19p13.1 | yes | ALCL |
| TPR | translocated promoter region | 7175 | 1 | 1q25 | yes | papillary thyroid |
| TSC1 | tuberous sclerosis 1 gene | 7248 | 9 | 9q34 | yes | renal cell carcinoma, bladder carcinoma |
| TSC2 | tuberous sclerosis 2 gene | 7249 | 16 | 16p13.3 | yes | pulmonary lymphangioleiomyomatosis (LAM), renal angiomyolipoma and head and neck cancer |
| TSHR | thyroid stimulating hormone receptor | 7253 | 14 | 14q31 | yes | toxic thyroid adenoma |
| U2AF1 | U2 small nuclear RNA auxiliary factor 1 | 7307 | 21 | 21q22.3 | yes | CLL, MDS |
| KDM6A | lysine (K)-specific demethylase 6A, ***UTX*** | 7403 | X | Xp11.2 | yes | renal, oesophageal SCC, MM |
| VHL | von Hippel-Lindau syndrome gene | 7428 | 3 | 3p25 | yes | renal, haemangioma, pheochromocytoma |
| EZR | ezrin | 7430 | 6 | 6q25.3 | yes | NSCLC |
| WAS | Wiskott-Aldrich syndrome | 7454 | X | Xp11.23-p11.22 |  |  |
| WHSC1 | Wolf-Hirschhorn syndrome candidate 1(MMSET) | 7468 | 4 | 4p16.3 | yes | MM |
| WRN | Werner syndrome (RECQL2) | 7486 | 8 | 8p12-p11.2 |  |  |
| WT1 | Wilms tumour 1 gene | 7490 | 11 | 11p13 | yes | Wilms tumour, desmoplastic small round cell tumour |
| XPA | xeroderma pigmentosum, complementation group A | 7507 | 9 | 9q22.3 |  |  |
| XPC | xeroderma pigmentosum, complementation group C | 7508 | 3 | 3p25 |  |  |
| XPO1 | exportin 1 (CRM1 homolog, yeast) | 7514 | 2 | 2p15 | yes | CLL |
| YWHAE | tyrosine 3-monooxygenase/tryptophan 5-monooxygenase activation protein, epsilon polypeptide (14-3-3 epsilon) | 7531 | 17 | 17p13.3 | yes | edometrial stromal sarcoma |
| ZNF9 | zinc finger protein 9 (a cellular retroviral nucleic acid binding protein) | 7555 | 3 | 3q21 | yes | aneurysmal bone cyst |
| ZNF145 | zinc finger protein 145 (PLZF) | 7704 | 11 | 11q23.1 | yes | APL |
| ZNF198 | zinc finger protein 198 | 7750 | 13 | 13q11-q12 | yes | MPD, NHL |
| PAX8 | paired box gene 8 | 7849 | 2 | 2q12-q14 | yes | follicular thyroid |
| DEK | DEK oncogene (DNA binding) | 7913 | 6 | 6p23 | yes | AML |
| TFEB | transcription factor EB | 7942 | 6 | 6p21 | yes | renal cell carcinoma (childhood epithelioid) |
| STL | Six-twelve leukemia gene | 7955 | 6 | 6q23 | yes | B-ALL |
| RUNXBP2 | runt-related transcription factor binding protein 2 (MOZ/ZNF220) | 7994 | 8 | 8p11 | yes | AML |
| NR4A3 | nuclear receptor subfamily 4, group A, member 3 (NOR1) | 8013 | 9 | 9q22 | yes | extraskeletal myxoid chondrosarcoma |
| BRD3 | bromodomain containing 3 | 8019 | 9 | 9q34 | yes | lethal midline carcinoma of young people |
| NUP214 | nucleoporin 214kDa (CAN) | 8021 | 9 | 9q34.1 | yes | AML, T-ALL |
| MLLT10 | myeloid/lymphoid or mixed-lineage leukemia (trithorax homolog, Drosophila); translocated to, 10 (AF10) | 8028 | 10 | 10p12 | yes | AL |
| CCDC6 | coiled-coil domain containing 6 | 8030 | 10 | 10q21 | yes | NSCLC |
| NCOA4 | nuclear receptor coactivator 4 - PTC3 (ELE1) | 8031 | 10 | 10q11.2 | yes | papillary thyroid |
| MLL2 | myeloid/lymphoid or mixed-lineage leukemia 2 | 8085 | 12 | 12q12-q14 | yes | medulloblastoma, renal |
| HMGA2 | high mobility group AT-hook 2 (HMGIC) | 8091 | 12 | 12q15 | yes | lipoma, leiomyoma, pleomorphic salivary gland adenoma |
| TCL1A | T-cell leukemia/lymphoma 1A | 8115 | 14 | 14q32.1 | yes | T-CLL |
| TAF15 | TAF15 RNA polymerase II, TATA box binding protein (TBP)-associated factor, 68kDa | 8148 | 17 | 17q11.1-q11.2 | yes | extraskeletal myxoid chondrosarcoma, ALL |
| ELL | ELL gene (11-19 lysine-rich leukemia gene) | 8178 | 19 | 19p13.1 | yes | AL |
| CLTCL1 | clathrin, heavy polypeptide-like 1 | 8218 | 22 | 22q11.21 | yes | ALCL |
| ZRSR2 | zinc finger (CCCH type), RNA-binding motif and serine/arginine rich 2 | 8233 | X | Xp22.1 | yes | MDS, CLL |
| KDM5C | lysine (K)-specific demethylase 5C ***(JARID1C***) | 8242 | X | Xp11.22-p11.21 | yes | clear cell renal carcinoma |
| ARID1A | AT rich interactive domain 1A (SWI-like) | 8289 | 1 | 1p35.3 | yes | clear cell ovarian carcinoma, RCC |
| HIST1H4I | histone 1, H4i (H4FM) | 8294 | 6 | 6p21.3 | yes | NHL |
| PICALM | phosphatidylinositol binding clathrin assembly protein (CALM) | 8301 | 11 | 11q14 | yes | TALL, AML, |
| AXIN1 | axin 1 | 8312 | 16 | 16p13.3 | yes | colorectal, endometrial, prostate and hepatocellular carcinomas, hepatoblastoma, sporadic medulloblastoma |
| BAP1 | BRCA1 associated protein-1 (ubiquitin carboxy-terminal hydrolase) | 8314 | 3 | 3p21.31-p21.2 | yes | uveal melanoma, breast, NSCLC, RCC |
| HIST1H3B | histone cluster 1, H3b | 8358 | 6 | 6p22.1 | yes | glioma |
| GAS7 | growth arrest-specific 7 | 8522 | 17 | 17p | yes | AML* |
| NCOA1 | nuclear receptor coactivator 1 | 8648 | 2 | 2p23 | yes | alveolar rhabdomyosarcoma |
| SOCS1 | suppressor of cytokine signaling 1 | 8651 | 16 | 16p13.13 | yes | Hodgkin lymphoma, PMBL |
| TNFRSF14 | tumor necrosis factor receptor superfamily, member 14 (herpesvirus entry mediator) | 8764 | 1 | 1p36.32 | yes | follicular lymphoma |
| TIF1 | transcriptional intermediary factor 1 (PTC6,TIF1A) | 8805 | 7 | 7q32-q34 | yes | APL |
| GMPS | guanine monphosphate synthetase | 8833 | 3 | 3q24 | yes | AML |
| FUBP1 | far upstream element (FUSE) binding protein 1 | 8880 | 1 | 1p13.1 | yes | oligodendroglioma |
| BCL10 | B-cell CLL/lymphoma 10 | 8915 | 1 | 1p22 | yes | MALT |
| PHOX2B | paired-like homeobox 2b | 8929 | 4 | 4p12 | yes | neuroblastoma |
| USP6 | ubiquitin specific peptidase 6 (Tre-2 oncogene) | 9098 | 17 | 17p13 | yes | aneurysmal bone cyst |
| RAB5EP | rabaptin, RAB GTPase binding effector protein 1 (RABPT5) | 9135 | 17 | 17p13 | yes | CMML |
| PCSK7 | proprotein convertase subtilisin/kexin type 7 | 9159 | 11 | 11q23.3 | yes | MLCLS |
| KLF4 | Kruppel-like factor 4 | 9314 | 9 | 9q31 | yes | meningioma |
| TRIP11 | thyroid hormone receptor interactor 11 | 9321 | 14 | 14q31-q32 | yes | AML |
| RECQL4 | RecQ protein-like 4 | 9401 | 8 | 8q24.3 |  |  |
| PDE4DIP | phosphodiesterase 4D interacting protein (myomegalin) | 9659 | 1 | 1q12 | yes | MPD |
| HERPUD1 | homocysteine-inducible, endoplasmic reticulum stress-inducible, ubiquitin-like domain member 1 | 9709 | 16 | 16q12.2-q13 | yes | prostate |
| SRGAP3 | SLIT-ROBO Rho GTPase activating protein 3 | 9901 | 3 | 3p25.3 | yes | pilocytic astrocytoma |
| MAFB | v-maf musculoaponeurotic fibrosarcoma oncogene homolog B (avian) | 9935 | 20 | 20q11.2-q13.1 | yes | MM |
| GOLGA5 | golgi autoantigen, golgin subfamily a, 5 (PTC5) | 9950 | 14 | 14q | yes | papillary thyroid |
| THRAP3 | thyroid hormone receptor associated protein 3 (TRAP150) | 9967 | 1 | 1p34.3 | yes | aneurysmal bone cyst |
| MED12 | mediator complex subunit 12 | 9968 | X | Xq13 | yes | uterine leiomyoma |
| SSH3BP1 | spectrin SH3 domain binding protein 1 | 10006 | 10 | 10p11.2 | yes | AML |
| SH2B3 | SH2B adaptor protein 3 | 10019 | 12 | 12q24.12 | yes | MPD, sAML, erythrocytosis, B-ALL |
| AKAP9 | A kinase (PRKA) anchor protein (yotiao) 9 | 10142 | 7 | 7q21-q22 | yes | papillary thyroid |
| LHFP | lipoma HMGIC fusion partner | 10186 | 13 | 13q12 | yes | lipoma |
| OLIG2 | oligodendrocyte lineage transcription factor 2 (BHLHB1) | 10215 | 21 | 21q22.11 | yes | T-ALL |
| GPHN | gephyrin (GPH) | 10243 | 14 | 14q24 | yes | AL |
| FSTL3 | follistatin-like 3 (secreted glycoprotein) | 10272 | 19 | 19p13 | yes | B-CLL |
| IKZF1 | IKAROS family zinc finger 1 | 10320 | 7 | 7p12.2 | yes | ALL, DLBCL |
| TFG | TRK-fused gene | 10342 | 3 | 3q11-q12 | yes | papillary thyroid, ALCL, NSCLC |
| NDRG1 | N-myc downstream regulated 1 | 10397 | 8 | 8q24.3 | yes | prostate |
| NCOA2 | nuclear receptor coactivator 2 (TIF2) | 10499 | 8 | 8q13.1 | yes | AML, chondrosarcoma |
| SLC34A2 | solute carrier family 34 (sodium phosphate), member 2 | 10568 | 4 | 4p15.2 | yes | NSCLC |
| MSF | MLL septin-like fusion | 10801 | 17 | 17q25 | yes | AML* |
| MALT1 | mucosa associated lymphoid tissue lymphoma translocation gene 1 | 10892 | 18 | 18q21 | yes | MALT |
| AF1Q | ALL1-fused gene from chromosome 1q | 10962 | 1 | 1q21 | yes | ALL |
| HEAB | ATP_GTP binding protein | 10978 | 11 | 11q12 | yes | AML |
| CEP1 | centrosomal protein 1 | 11064 | 9 | 9q33 | yes | MPD, NHL |
| FGFR1OP | FGFR1 oncogene partner (FOP) | 11116 | 6 | 6q27 | yes | MPD, NHL |
| PSIP2 | PC4 and SFRS1 interacting protein 2 (LEDGF) | 11168 | 9 | 9p22.2 | yes | AML |
| WIF1 | WNT inhibitory factor 1 | 11197 | 12 | 12q14.3 | yes | pleomorphic salivary gland adenoma |
| CHEK2 | CHK2 checkpoint homolog (S. pombe) | 11200 | 22 | 22q12.1 |  |  |
| DUX4 | double homeobox, 4 | 22947 | 4 | 4q35 | yes | soft tissue sarcoma |
| NT5C2 | 5'-nucleotidase, cytosolic II | 22978 | 10 | 10q24.32 | yes | relapse ALL |
| FNBP1 | formin binding protein 1 (FBP17) | 23048 | 9 | 9q23 | yes | AML |
| ELKS | ELKS protein | 23085 | 12 | 12p13.3 | yes | papillary thyroid |
| GRAF | GTPase regulator associated with focal adhesion kinase pp125(FAK) | 23092 | 5 | 5q31 | yes | AML, MDS |
| CIC | capicua homolog | 23152 | 19 | 19q13.2 | yes | oligodendroglioma, soft tissue sarcoma |
| SEPT6 | septin 6 | 23157 | X | Xq24 | yes | AML |
| CAMTA1 | calmodulin binding transcription activator 1 | 23261 | 1 | 1p36.31-p36.23 | yes | epithelioid haemangioendothelioma |
| FACL6 | fatty-acid-coenzyme A ligase, long-chain 6 | 23305 | 5 | 5q31 | yes | AML, AEL |
| ARHGEF12 | RHO guanine nucleotide exchange factor (GEF) 12 (LARG) | 23365 | 11 | 11q23.3 | yes | AML |
| MECT1 | mucoepidermoid translocated 1 | 23373 | 19 | 19p13 | yes | salivary gland mucoepidermoid |
| DICER1 | dicer 1, ribonuclease type III | 23405 | 14 | 14q32.13 | yes | sex cord-stromal tumour, TGCT, embryonal rhabdomyosarcoma |
| SF3B1 | splicing factor 3b, subunit 1, 155kDa | 23451 | 2 | 2q33.1 | yes | myelodysplastic syndrome |
| HEY1 | hairy/enhancer-of-split related with YRPW motif 1 | 23462 | 8 | 8q21 | yes | mesenchymal chondrosarcoma |
| BRD4 | bromodomain containing 4 | 23476 | 19 | 19p13.1 | yes | lethal midline carcinoma of young people |
| SUZ12 | suppressor of zeste 12 homolog (Drosophila) | 23512 | 17 | 17q11.2 | yes | endometrial stromal tumour |
| MYST4 | MYST histone acetyltransferase (monocytic leukemia) 4 (MORF) | 23522 | 10 | 10q22 | yes | AML |
| ZNF278 | zinc finger protein 278 (ZSG) | 23598 | 22 | 22q12-q14 | yes | Ewing sarcoma |
| CBLC | Cas-Br-M (murine) ecotropic retroviral transforming sequence c | 23624 | 19 | 19q13.2 | yes | AML |
| POT1 | protection of telomeres 1 | 25913 | 7 | 7q31.33 | yes | CLL |
| ZNF521 | zinc finger protein 521 | 25925 | 18 | 18q11.2 | yes | ALL |
| SS18L1 | synovial sarcoma translocation gene on chromosome 18-like 1 | 26039 | 20 | 20q13.3 | yes | synovial sarcoma |
| SETBP1 | SET binding protein 1 | 26040 | 18 | 18q21.1 | yes | atypical CML |
| CHIC2 | cysteine-rich hydrophobic domain 2 | 26511 | 4 | 4q11-q12 | yes | AML |
| TCL6 | T-cell leukemia/lymphoma 6 | 27004 | 14 | 14q32.1 | yes | T-ALL |
| FOXP1 | forkhead box P1 | 27086 | 3 | 3p14.1 | yes | ALL |
| AF5q31 | ALL1 fused gene from 5q31 | 27125 | 5 | 5q31 | yes | ALL |
| EML4 | echinoderm microtubule associated protein like 4 | 27436 | 2 | 2p21 | yes | NSCLC |
| PRO1073 | PRO1073 protein (ALPHA) | 29005 | 11 | 11q31.1 | yes | renal cell carcinoma (childhood epithelioid) |
| SETD2 | SET domain containing 2 | 29072 | 3 | 3p21.31 | yes | clear cell renal carcinoma |
| CD274 | CD274 molecule | 29126 | 9 | 9p24 | yes | PMBL, Hodgkin lymphoma |
| TFPT | TCF3 (E2A) fusion partner (in childhood leukaemia) | 29844 | 19 | 19q13 | yes | pre-B ALL |
| TLX3 | T-cell leukemia, homeobox 3 (HOX11L2) | 30012 | 5 | 5q35.1 | yes | T-ALL |
| IL21R | interleukin 21 receptor | 50615 | 16 | 16p11 | yes | NHL |
| IGK@ | immunoglobulin kappa locus | 50802 | 2 | 2p12 | yes | Burkitt lymphoma, B-NHL |
| SBDS | Shwachman-Bodian-Diamond syndrome protein | 51119 | 7 | 7q11 |  |  |
| NIN | ninein (GSK3B interacting protein) | 51199 | 14 | 14q24 | yes | MPD |
| AF3p21 | SH3 protein interacting with Nck, 90 kDa (ALL1 fused gene from 3p21) | 51517 | 3 | 3p21 | yes | ALL |
| TRIM33 | tripartite motif-containing 33 (PTC7,TIF1G) | 51592 | 1 | 1p13 | yes | papillary thyroid |
| SUFU | suppressor of fused homolog (Drosophila) | 51684 | 10 | 10q24.32 | yes | medulloblastoma |
| CDK12 | cyclin-dependent kinase 12 | 51755 | 17 | 17q12 | yes | serous ovarian |
| BCL11A | B-cell CLL/lymphoma 11A | 53335 | 2 | 2p13 | yes | B-CLL |
| FEV | FEV protein - (HSRNAFEV) | 54738 | 2 | 2q36 | yes | Ewing sarcoma |
| TET2 | tet oncogene family member 2 | 54790 | 4 | 4q24 | yes | MDS |
| FAM46C | family with sequence similarity 46, member C | 54855 | 1 | 1p12 | yes | MM |
| BCOR | BCL6 corepressor | 54880 | X | Xp11.4 | yes | retinoblastoma, AML, APL (translocation) |
| RNF43 | Ring finger protein 43 | 54894 | 17 | 17q22 | yes | cholangiocarcinoma, ovary, pancreas |
| WHSC1L1 | Wolf-Hirschhorn syndrome candidate 1-like 1 (NSD3) | 54904 | 8 | 8p12 | yes | AML |
| SDH5 | chromosome 11 open reading frame 79 | 54949 | 11 | 11q12.2 |  |  |
| PBRM1 | polybromo 1 | 55193 | 3 | 3p21 | yes | clear cell renal carcinoma, breast |
| FBXW7 | F-box and WD-40 domain protein 7 (archipelago homolog, Drosophila) | 55294 | 4 | 4q31.3 | yes | colorectal, endometrial, T-ALL |
| ZNF331 | zinc finger protein 331 | 55422 | 19 | 19q13.3-q13.4 | yes | follicular thyroid adenoma |
| CMKOR1 | chemokine orphan receptor 1 | 57007 | 2 | 2q37.3 | yes | lipoma |
| AF15Q14 | AF15q14 protein | 57082 | 15 | 15q14 | yes | AML |
| GOPC | golgi associated PDZ and coiled-coil motif containing | 57120 | 6 | 6q21 | yes | glioblastoma |
| MKL1 | megakaryoblastic leukemia (translocation) 1 | 57591 | 22 | 22q13 | yes | acute megakaryocytic leukaemia |
| KIAA1549 | KIAA1549 | 57670 | 7 | 7q34 | yes | pilocytic astrocytoma |
| ALO17 | KIAA1618 protein | 57674 | 17 | 17q25.3 | yes | ALCL |
| CCNB1IP1 | cyclin B1 interacting protein 1, E3 ubiquitin protein ligase | 57820 | 14 | 14q11.2 | yes | leiomyoma |
| MLL3 | myeloid/lymphoid or mixed-lineage leukemia 3 | 58508 | 7 | 7q36.1 | yes | medulloblastoma |
| PRDM16 | PR domain containing 16 | 63976 | 1 | 1p36.23-p33 | yes | MDS, AML |
| CRLF2 | cytokine receptor-like factor 2 | 64109 | X,Y | Xp22.3; Yp11.3 | yes | B-ALL, Downs associated ALL |
| NSD1 | nuclear receptor binding SET domain protein 1 | 64324 | 5 | 5q35 | yes | AML |
| CREB3L2 | cAMP responsive element binding protein 3-like 2 | 64764 | 7 | 7q34 | yes | fibromyxoid sarcoma |
| RBM15 | RNA binding motif protein 15 | 64783 | 1 | 1p13 | yes | acute megakaryocytic leukaemia |
| CRTC3 | CREB regulated transcription coactivator 3 | 64784 | 15 | 15q26.1 | yes | salivary gland mucoepidermoid |
| RANBP17 | RAN binding protein 17 | 64901 | 5 | 5q34 | yes | ALL |
| BCL11B | B-cell CLL/lymphoma 11B (CTIP2) | 64919 | 14 | 14q32.1 | yes | T-ALL |
| ASPSCR1 | alveolar soft part sarcoma chromosome region, candidate 1 | 79058 | 17 | 17q25 | yes | alveolar soft part sarcoma |
| CHCHD7 | coiled-coil-helix-coiled-coil-helix domain containing 7 | 79145 | 8 | 8q11.2 | yes | salivary gland adenoma |
| PALB2 | partner and localizer of BRCA2 | 79728 | 16 | 16p12.1 |  |  |
| FBXO11 | F-box protein 11 | 80204 | 2 | 2p16.3 | yes | DLBCL |
| C2orf44 | chromosome 2 open reading frame 44 | 80304 | 2 | 2p23.3 | yes | NSCLC |
| LCX | leukemia-associated protein with a CXXC domain | 80312 | 10 | 10q21 | yes | AML |
| CD273 | programmed cell death 1 ligand 2 | 80380 | 9 | 9p24.2 | yes | PMBL, Hodgkin lymphoma |
| FIP1L1 | FIP1 like 1 (S. cerevisiae) | 81608 | 4 | 4q12 | yes | idiopathic hypereosinophilic syndrome |
| IRTA1 | immunoglobulin superfamily receptor translocation associated 1 | 83417 | 1 | 1q21 | yes | B-NHL |
| BRIP1 | BRCA1 interacting protein C-terminal helicase 1 | 83990 | 17 | 17q22 |  |  |
| RUNDC2A | RUN domain containing 2A | 84127 | 16 | 16p13.13 | yes | PMBL, Hodgkin lymphoma |
| TRAF7 | tumour necrosis factor receptor-associated factor 7 | 84231 | 16 | 16p13.3 | yes | meningioma |
| PHF6 | PHD finger protein 6 | 84295 | X | Xq26.3 | yes | ETP ALL |
| HOOK3 | hook homolog 3 | 84376 | 8 | 8p11.21 | yes | papillary thyroid |
| CARD11 | caspase recruitment domain family, member 11 | 84433 | 7 | 7p22 | yes | DLBCL |
| MAML2 | mastermind-like 2 (Drosophila) | 84441 | 11 | 11q22-q23 | yes | salivary gland mucoepidermoid |
| SLC45A3 | solute carrier family 45, member 3 | 85414 | 1 | 1q32 | yes | prostate |
| CREB3L1 | cAMP responsive element binding protein 3-like 1 | 90993 | 11 | 11p11.2 | yes | myxofibrosarcoma |
| HCMOGT-1 | sperm antigen HCMOGT-1 | 92521 | 17 | 17p11.2 | yes | JMML |
| C12orf9 | chromosome 12 open reading frame 9 | 93669 | 12 | 12q14.3 | yes | lipoma |
| C16orf75 | chromosome 16 open reading frame 75 | 116028 | 16 | 16p13.13 | yes | PMBL, Hodgkin lymphoma |
| LRIG3 | leucine-rich repeats and immunoglobulin-like domains 3 | 121227 | 12 | 12q14.1 | yes | NSCLC |
| MSI2 | musashi homolog 2 (Drosophila) | 124540 | 17 | 17q23.2 | yes | CML |
| CANT1 | calcium activated nucleotidase 1 | 124583 | 17 | 17q25 | yes | prostate |
| WTX | family with sequence similarity 123B (FAM123B) | 139285 | X | Xq11.1 | yes | Wilms tumour |
| VTI1A | vesicle transport through interaction with t-SNAREs homolog 1A | 143187 | 10 | 10q25.2 | yes | colorectal |
| FLJ27352 | BX648577, FLJ27352 hypothetical LOC145788 | 145788 | 15 | 15q21.3 | yes | PMBL, Hodgkin lymphoma |
| TTL | tubulin tyrosine ligase | 150465 | 2 | 2q13 | yes | ALL |
| ZNF384 | zinc finger protein 384 (CIZ/NMP4) | 171017 | 12 | 12p13 | yes | ALL |
| ASXL1 | additional sex combs like 1 | 171023 | 20 | 20q11.1 | yes | MDS, CMML |
| ARID2 | AT rich interactive domain 2 | 196528 | 12 | 12q12 | yes | hepatocellular carcinoma |
| BHD | folliculin, Birt-Hogg-Dube syndrome | 201163 | 17 | 17p11.2 |  |  |
| JAZF1 | juxtaposed with another zinc finger gene 1 | 221895 | 7 | 7p15.2-p15.1 | yes | endometrial stromal tumour |
| C15orf55 | chromosome 15 open reading frame 55 | 256646 | 15 | 15q14 | yes | lethal midline carcinoma |
| MDS2 | myelodysplastic syndrome 2 | 259283 | 1 | 1p36 | yes | MDS |
| C15orf21 | chromosome 15 open reading frame 21 | 283651 | 15 | 15q21.1 | yes | prostate |
| P2RY8 | purinergic receptor P2Y, G-protein coupled, 8 | 286530 | X,Y | Xp22.3; Yp11.3 | yes | B-ALL, Down syndrome associated ALL |
| ECT2L | epithelial cell transforming sequence 2 oncogene-like | 345930 | 6 | 6q24.1 | yes | ETP ALL |
| WWTR1 | WW domain containing transcription regulator 1 | 607392 | 3 | 3q23-q24 | yes | epithelioid haemangioendothelioma |
| FAM22A | family with sequence similarity 22, member A | 728118 | 10 | 10q23.2 | yes | endometrial stromal sarcoma |
| FAM22B | family with sequence similarity 22, member B | 729262 | 10 | 10q22.3 | yes | endometrial stromal sarcoma |
