# Supplementary material for: Identification of MAP kinase pathways as therapeutic targets in gallbladder carcinoma using targeted parallel sequencing
Source: Oncotarget. 2017 Mar 31;8(22):36319–30. doi: 10.18632/oncotarget.16751 (PMC5482657; doi:10.18632/oncotarget.16751)
Supplement: Supplementary file 3 [file oncotarget-08-36319-s003.docx]

**Supplementary Table 3:** **Recurrent mutations identified in this study**

| **Symbol** | **Chromosome** | **Mutated Samples** | **Gene Region** | **SIFT Score** | **Function** | **Prtedicted Protein Variants** | **Mutation rate** |
| --- | --- | --- | --- | --- | --- | --- | --- |
| ABL1 | 9 | T4 | Exonic | 0 | Nonsynonymous SNV | NM_007313:exon3:c.385T>G:p.W129G;NM_005157:exon3:c.328T>G:p.W110G | 7.10% |
| ADAM12 | 10 | T7 | Splicing |  | Splicing mutation | NM_003474,NM_021641:exon16:c.1619-7G>C | 7.10% |
| AFF3 | 2 | T7,T17,T31 | Exonic |  | Nonframeshift deletion | NM_002285:exon14:c.1783_1788del:p.595_596del;NM_001025108:exon14:c.1858_1863del:p.620_621del | 21.40% |
|  | 2 |  | Exonic | 0.39 | Nonsynonymous SNV | NM_002285:exon14:c.2249G>A:p.R750Q;NM_001025108:exon14:c.2324G>A:p.R775Q |  |
|  | 2 |  | Exonic | 0.15 | Nonsynonymous SNV | NM_002285:exon14:c.1781C>G:p.T594S;NM_001025108:exon14:c.1856C>G:p.T619S |  |
| AIM1 | 6 | T9 | Exonic | 0.04 | Nonsynonymous SNV | NM_001624:exon16:c.4598C>T:p.T1533I | 7.10% |
| APC | 5 | T14 | Exonic |  | Frameshift deletion | NM_000038,NM_001127510:exon16:c.4385_4388del:p.1462_1463del;NM_001127511:exon14:c.4331_4334del:p.1444_1445del | 7.10% |
| ARID1A | 1 | T34,T36 | Exonic |  | Frameshift insertion | NM_139135:exon20:c.4890_4891insG:p.G1630fs,ARID1A:NM_006015:exon20:c.5541_5542insG:p.G1847fs, | 14.30% |
|  | 1 |  | Exonic |  | Frameshift insertion | NM_139135:exon20:c.5299_5300insT:p.V1767fs;NM_006015:exon20:c.5950_5951insT:p.V1984fs |  |
| ARID2 | 12 | T4 | Exonic | 0 | Nonsynonymous SNV | NM_152641:exon17:c.4940C>T:p.S1647L | 7.10% |
|  | 12 |  | Exonic;splicing | 0.31 | Stopgain SNV | NM_152641:exon17:c.4930C>T:p.Q1644X |  |
| ATP10B | 5 | T14 | Exonic | 0.65 | Nonsynonymous SNV | NM_025153:exon23:c.3578C>T:p.S1193L | 7.10% |
| ATP13A3 | 3 | T9 | Exonic | 0.15 | Nonsynonymous SNV | NM_024524:exon4:c.325G>A:p.G109S | 7.10% |
| BCL6 | 3 | T30 | Exonic | 0.09 | Nonsynonymous SNV | NM_001130845:exon5:c.974G>A:p.R325Q | 7.10% |
| BRCA1 | 17 | T9 | Exonic | 0.7 | Nonsynonymous SNV | NM_007300,NM_007294:exon10:c.1036C>T:p.P346S;NM_007297:exon9:c.895C>T:p.P299S | 7.10% |
| BRD4 | 19 | T7 | Exonic | 0.27 | Nonsynonymous SNV | NM_014299,NM_058243:exon5:c.802G>A:p.V268I | 7.10% |
| CARD10 | 22 | T7 | Exonic | 0 | Nonsynonymous SNV | NM_014550:exon20:c.3077G>A:p.C1026Y | 7.10% |
| CARD11 | 7 | T9 | Splicing |  | Splicing mutation | NM_032415:exon25:c.3260+3A>G | 7.10% |
| CASC5 | 15 | T34 | Exonic | 0.16 | Nonsynonymous SNV | NM_170589:exon18:c.6121A>C:p.M2041L;NM_144508:exon17:c.6043A>C:p.M2015L | 7.10% |
| CBL | 11 | T4 | Exonic | 0.01 | Nonsynonymous SNV | NM_005188:exon11:c.1930G>C:p.D644H | 7.10% |
| CCND1 | 11 | T9 | Exonic;splicing | 0 | Nonsynonymous SNV | NM_053056:exon4:c.718G>A:p.D240N | 7.10% |
| CDC6 | 17 | T4 | Exonic | 0.03 | Nonsynonymous SNV | NM_001254:exon4:c.487G>C:p.V163L | 7.10% |
| CDH10 | 5 | T4 | Exonic | 0.25 | Nonsynonymous SNV | NM_006727:exon11:c.1819G>A:p.A607T | 7.10% |
| CDKN2A | 9 | T9,T13 | Exonic | 0 | Stopgain SNV | NM_001195132,NM_058197,NM_000077:exon1:c.35C>A:p.S12X | 14.30% |
|  | 9 |  | Splicing |  | Splicing mutation | NM_001195132:exon5:c.655-1G>C;NM_058197:exon4:c.732-1G>C;NM_000077:exon4:c.458-1G>C;NM_058195:exon4:c.501-1G>C |  |
| CLTC | 17 | T4 | Exonic | 0.05 | Nonsynonymous SNV | NM_004859:exon9:c.1396C>G:p.L466V | 7.10% |
| CTNNA1 | 5 | T9 | Exonic | 0.01 | Stopgain SNV | NM_001903:exon3:c.265G>T:p.E89X | 7.10% |
| CTNNB1 | 3 | T16,T17 | Exonic | 0 | Nonsynonymous SNV | NM_001904,NM_001098210,NM_001098209:exon3:c.134C>T:p.S45F | 14.30% |
| ERBB2 | 17 | T9 | Splicing |  | Splicing mutation | NM_001005862:exon11:c.812-6C>T;NM_004448:exon8:c.902-6C>T | 7.10% |
| ETV4 | 17 | T7 | Exonic;splicing | 0 | Stopgain SNV | NM_001079675,NM_001986:exon8:c.804C>G:p.Y268X;NM_001261437,NM_001261438:exon7:c.687C>G:p.Y229X | 7.10% |
| FBXW7 | 4 | T13 | Exonic | 0 | Nonsynonymous SNV | NM_018315:exon8:c.1153C>T:p.R385C;NM_001013415:exon8:c.1039C>T:p.R347C;NM_033632:exon9:c.1393C>T:p.R465C | 7.10% |
| GLI3 | 7 | T9 | Exonic | 0.18 | Nonsynonymous SNV | NM_000168:exon15:c.3908A>T:p.E1303V | 7.10% |
| GRIN2A | 16 | T9 | Exonic;splicing | 0.12 | Nonsynonymous SNV | NM_001134407,NM_000833,NM_001134408:exon10:c.2165C>T:p.T722M | 7.10% |
| IGFBP3 | 7 | T9 | Exonic | 0.25 | Nonsynonymous SNV | NM_000598,NM_001013398:exon1:c.95C>G:p.A32G | 7.10% |
| INF2 | 14 | T15,T17 | Exonic |  | Nonframeshift deletion | NM_001031714,NM_022489:exon8:c.1259_1270del:p.420_424del | 14.30% |
| IRAK2 | 3 | T9 | Exonic | 0.01 | Nonsynonymous SNV | NM_001570:exon12:c.1511G>A:p.R504Q | 7.10% |
| IRF8 | 16 | T9 | Exonic | 0.02 | Nonsynonymous SNV | NM_002163:exon7:c.850C>T:p.R284W | 7.10% |
| JAZF1 | 7 | T5 | Splicing |  | Splicing mutation | NM_175061:exon2:c.115+8G>C | 7.10% |
| KDM5C | X | T7 | Exonic | 0.05 | Nonsynonymous SNV | NM_001282622:exon17:c.2489G>T:p.G830V;NM_004187:exon17:c.2492G>T:p.G831V;NM_001146702:exon15:c.2291G>T:p.G764V | 7.10% |
| KIAA1549 | 7 | T9 | Exonic | 0.02 | Nonsynonymous SNV | NM_020910,NM_001164665:exon2:c.2710G>A:p.A904T | 7.10% |
| KMT2C | 7 | T4 | Exonic | 1 | Stopgain SNV | NM_170606:exon36:c.6082C>T:p.R2028X | 7.10% |
| KMT2D | 12 | T4 | Exonic |  | Stopgain SNV | NM_003482:exon52:c.16360C>T:p.R5454X | 7.10% |
|  | 12 |  | Exonic |  | Stopgain SNV | NM_003482:exon31:c.7264C>T:p.Q2422X |  |
| KRAS | 12 | T16 | Exonic | 0.01 | Nonsynonymous SNV | NM_033360,NM_004985:exon3:c.183A>T:p.Q61H | 7.10% |
| LYL1 | 19 | T7 | Exonic | 0.01 | Nonsynonymous SNV | NM_005583:exon3:c.368T>C:p.I123T | 7.10% |
| MAP2K1 | 15 | T9 | Exonic | 0.03 | Nonsynonymous SNV | NM_002755:exon7:c.715C>T:p.H239Y | 7.10% |
| MAPKBP1 | 15 | T9 | Exonic | 0.32 | Nonsynonymous SNV | NM_001128608:exon30:c.3722C>T:p.P1241L;NM_014994:exon29:c.3704C>T:p.P1235L | 7.10% |
| MAX | 14 | T36 | Exonic | 0.01 | Nonsynonymous SNV | NM_145112:exon3:c.202A>G:p.K68E;NM_002382,NM_145113:exon4:c.229A>G:p.K77E | 7.10% |
| MLH1 | 3 | T9 | Exonic | 0 | Nonsynonymous SNV | NM_000249,NM_001258271:exon8:c.649C>T:p.R217C;NM_001167617:exon8:c.355C>T:p.R119C | 7.10% |
| MLLT10 | 10 | T7 | Exonic | 0.01 | Nonsynonymous SNV | NM_004641:exon21:c.2891C>G:p.A964G;NM_001195626:exon19:c.2843C>G:p.A948G | 7.10% |
| MTOR | 1 | T7 | Exonic | 1 | Nonsynonymous SNV | NM_004958:exon20:c.3054G>T:p.M1018I | 7.10% |
| NACA | 12 | T9 | Exonic | 0 | Nonsynonymous SNV | NM_001113203:exon3:c.1747C>A:p.P583T | 7.10% |
| NF1 | 17 | T4,T16,T17 | Splicing |  | Splicing mutation | NM_001128147,NM_000267,NM_001042492:exon14:c.1641+1G>A | 21.40% |
|  | 17 |  | Splicing |  | Splicing mutation | NM_000267:exon52:c.7676-2A>T;NM_001042492:exon53:c.7739-2A>T |  |
|  | 17 |  | Exonic;splicing | 0 | Nonsynonymous SNV | NM_001042492,NM_001128147,NM_000267:exon15:c.1645C>G:p.L549V |  |
| NFE2L2 | 2 | T13 | Exonic | 0 | Nonsynonymous SNV | NM_001145413,NM_001145412:exon2:c.187G>A:p.E63K;NM_006164:exon2:c.235G>A:p.E79K, | 7.10% |
| NOTCH2 | 1 | T31 | Exonic | 0.29 | Nonsynonymous SNV | NM_024408:exon34:c.7195G>A:p.E2399K | 7.10% |
| PDCD11 | 10 | T16 | Exonic | 0.2 | Nonsynonymous SNV | NM_014976:exon18:c.2524G>A:p.E842K | 7.10% |
| PDGFRA | 4 | T7 | Exonic | 0 | Nonsynonymous SNV | NM_006206:exon17:c.2381A>G:p.D794G | 7.10% |
| PIK3CA | 3 | T16 | Exonic | 0 | Nonsynonymous SNV | NM_006218:exon10:c.1633G>A:p.E545K | 7.10% |
| POT1 | 7 | T9 | Exonic | 0.11 | Nonsynonymous SNV | NM_015450:exon7:c.191G>T:p.G64V | 7.10% |
| PPP1R13L | 19 | T16 | Exonic | 0 | Nonsynonymous SNV | NM_001142502,NM_006663:exon10:c.2026G>A:p.V676M | 7.10% |
| PRF1 | 10 | T13 | Exonic | 0.02 | Nonsynonymous SNV | NM_001083116,NM_005041:exon3:c.1390C>T:p.R464W | 7.10% |
| PSIP1 | 9 | T9 | Exonic | 0 | Nonsynonymous SNV | NM_033222,NM_001128217:exon13:c.1118G>A:p.C373Y | 7.10% |
| RANBP17 | 5 | T9 | Exonic | 0.04 | Nonsynonymous SNV | NM_022897:exon7:c.728G>A:p.C243Y | 7.10% |
| RECQL4 | 8 | T36 | Exonic | 0.08 | Nonsynonymous SNV | NM_004260:exon4:c.280C>A:p.P94T | 7.10% |
| RELA | 11 | T14 | Exonic | 0 | Stopgain SNV | NM_021975,NM_001243984,NM_001145138,NM_001243985:exon3:c.115G>T:p.E39X | 7.10% |
| RRAD | 16 | T30 | Splicing |  | Splicing mutation | NM_001128850,NM_004165:exon4:c.371-8C>T | 7.10% |
| SMAD1 | 4 | T13 | Exonic | 0 | Nonsynonymous SNV | NM_005900,NM_001003688:exon3:c.433G>A:p.E145K | 7.10% |
| SRSF3 | 6 | T7 | Exonic |  | Nonsynonymous SNV | NM_003017:exon3:c.314G>T:p.R105L | 7.10% |
| TLX1 | 10 | T7,T9 | Exonic | 0.1 | Nonsynonymous SNV | NM_001195517,NM_005521:exon1:c.37C>A:p.H13N | 14.30% |
|  | 10 |  | Exonic | 0.1 | Nonsynonymous SNV | NM_001195517,NM_005521:exon1:c.113C>T:p.S38L |  |
| TP53 | 17 | T7,T9,T13,T16,T30,T34,T36 | Exonic | 0 | Nonsynonymous SNV | NM_001276698:exon3:c.265C>T:p.R89W;NM_001126116:exon3:c.346C>T:p.R116W;NM_001126112,NM_000546,NM_001126114:exon7:c.742C>T:p.R248W;NM_001276761:exon7:c.625C>T:p.R209W | 50.00% |
|  | 17 |  | Exonic | 0 | Nonsynonymous SNV | NM_001276698,NM_001126116:exon4:c.340C>T:p.R114C;NM_001126112,NM_000546,NM_001126114:exon8:c.817C>T:p.R273C;NM_001276761:exon8:c.700C>T:p.R234C |  |
|  | 17 |  | Exonic | 0 | Nonsynonymous SNV | NM_001276698:exon2:c.182A>G:p.Y61C;NM_001126116:exon2:c.263A>G:p.Y88C;NM_001126112,NM_000546,NM_001126114:exon6:c.659A>G:p.Y220C;NM_001276761:exon6:c.542A>G:p.Y181C |  |
|  | 17 |  | Exonic | 0 | Nonsynonymous SNV | NM_001276698:exon1:c.47G>A:p.R16H;NM_001126116:exon1:c.128G>A:p.R43H;NM_001126112,NM_000546,NM_001126114:exon5:c.524G>A:p.R175H;NM_001276761:exon5:c.407G>A:p.R136H |  |
|  | 17 |  | Exonic | 0.04 | Stopgain SNV | NM_001276698:exon2:c.97C>T:p.Q33X;NM_001126116:exon2:c.178C>T:p.Q60X;NM_001126112,NM_001126114,NM_000546:exon6:c.574C>T:p.Q192X;NM_001276761:exon6:c.457C>T:p.Q153X |  |
|  | 17 |  | Exonic | 0 | Nonsynonymous SNV | NM_001276698:exon2:c.100C>T:p.H34Y;NM_001126116:exon2:c.181C>T:p.H61Y;NM_001126112,NM_000546,NM_001126114:exon6:c.577C>T:p.H193Y;NM_001276761:exon6:c.460C>T:p.H154Y |  |
|  | 17 |  | Exonic | 0 | Stopgain SNV | NM_001276698:exon1:c.12C>A:p.Y4X;NM_001126116:exon1:c.93C>A:p.Y31X;NM_001126112:exon5:c.489C>A:p.Y163X;NM_001276761:exon5:c.372C>A:p.Y124X;NM_000546,NM_001126114:exon5:c.489C>A:p.Y163X;:exon5:c.489C>A:p.Y163X |  |
|  | 17 |  | Exonic | 0 | Nonsynonymous SNV | NM_001276698:exon3:c.218T>G:p.I73S;NM_001126116:exon3:c.299T>G:p.I100S;NM_001126112,NM_001126114,NM_000546:exon7:c.695T>G:p.I232S;NM_001276761:exon7:c.578T>G:p.I193S |  |
|  | 17 |  | Exonic |  | Nonframeshift deletion | NM_001276698:exon1:c.13_15del:p.5_5del;NM_001126116:exon1:c.94_96del:p.32_32del;NM_001126112,NM_000546:exon5:c.490_492del:p.164_164del;NM_001276761:exon5:c.373_375del:p.125_125del |  |
| WHSC1 | 4 | T7 | Splicing |  | Splicing mutation | NM_001042424,NM_133335:exon5:c.928-6T>C;NM_007331,NM_133330:exon7:c.928-6T>C;NM_133331:exon6:c.928-6T>C;NM_133334:exon4:c.928-6T>C | 7.10% |
| ZNF521 | 18 | T9,30 | Exonic | 0.37 | Nonsynonymous SNV | NM_015461:exon4:c.1480G>A:p.E494K | 14.30% |
|  | 18 |  | Exonic | 1 | Nonsynonymous SNV | NM_015461:exon4:c.1387C>G:p.Q463E | 7.10% |
| ZRSR2 | X | T44 | Exonic | 0.36 | Nonsynonymous SNV | NM_005089:exon3:c.122G>A:p.G41E | 7.10% |
